# Ameliorating motor performance and quality of life in Parkinson’s disease: a comparison of deep brain stimulation and focused ultrasound surgery

**DOI:** 10.3389/fneur.2025.1449973

**Published:** 2025-04-30

**Authors:** Mingqian Liang, Le Hou, Jinjun Liang, Shengyong Bao

**Affiliations:** ^1^Department of Rehabilitation Medicine, Shenzhen People’s Hospital, The Second Clinical Medical College, Jinan University, The First Affiliated Hospital, Southern University of Science and Technology, Shenzhen, Guangdong, China; ^2^Psychological Sleep Department, The First Affiliated Hospital of Guangzhou University of Chinese Medicine (Guangdong Clinical Research Academy of Chinese Medicine), Guangzhou, Guangdong, China; ^3^Department of Rehabilitation Medicine, Zhongshan Sixth People’s Hospital (Torch Development Zone People’s Hospital), Zhongshan, Guangdong, China

**Keywords:** deep brain stimulation, magnetic resonance-guided focused ultrasound surgery, network meta-analysis, Parkinson’s disease, quality of life

## Abstract

**Introduction:**

Deep brain stimulation (DBS) and magnetic resonance-guided focused ultrasound surgery (MRgFUS) have emerged as valuable treatment options for Parkinson’s disease (PD) with drug-resistant symptoms. However, comparative studies of various DBS targets and MRgFUS are still limited.

**Methods:**

We reviewed three databases for trials on the effects of DBS or MRgFUS on PD patients, focusing on motor performance and quality of life (QoL). A frequentist network meta-analysis was conducted to estimate the treatment effects.

**Results:**

There were 39 trials in this study, comprising 3,002 patients. In the off-phase, subthalamic nucleus_DBS (STN_DBS [SMD, −0.94; 95%CI, −1.40 to −0.48]) significantly improved the UPDRS-III Total score compared to medication treatment alone (MT). In the on-phase, STN_DBS (SMD, −0.83; 95%CI, −1.13 to −0.53), internal globus pallidus_DBS (GPi_DBS [SMD, −0.80; 95%CI, −1.20 to −0.40]), and STN_Focused Ultrasound (STN_FUS [SMD, −1.83; 95%CI, −2.97 to −0.68]) significantly improved the UPDRS-III Total score. Regarding QoL, STN_DBS (SMD, −0.75; 95% CI, −1.46 to −0.05) and GPi_DBS (SMD, −0.58; 95% CI, −0.96 to −0.21) demonstrated better outcomes compared to MT. The SUCRA plot indicated that the top three treatments for UPDRS-III Total score in the off-phase were STN_FUS (79.6%), STN-GPi_DBS (73.7%), and STN_DBS (69.1%). In the on-phase, the top three treatments were STN_FUS (95.7%), STN_DBS (69.6%), and GPi_DBS (66.9%). Regarding QoL, GPi_DBS (77.2%) ranks first, followed by STN_DBS (67.3%), STN_FUS (56.9%) ranks third.

**Conclusion:**

STN_DBS, GPi_DBS, and STN_FUS have exhibited efficacy in ameliorating motor performance and enhancing QoL in PD patients. Nevertheless, as a potential alternative to STN_DBS with comparable efficacy, STN-FUS may serve as another treatment option.

## Introduction

Parkinson’s disease (PD) is a neurodegenerative disorder characterized by resting tremors, bradykinesia, rigidity, and postural disturbances typically progressing over time (1). As the most common movement disorder, PD currently affects approximately 6.2 million individuals, with the figure projected to double by 2040 (2, 3). As PD evolves, motor complications can appear and progressively worsen, substantially affecting not only the general quality of life (QoL) but also the daily routines of those afflicted.

Dopamine-based medications are essential for alleviating both motor and non-motor symptoms in individuals with PD (4). However, prolonged administration of these medications frequently gives rise to drug-induced dyskinesias and motor fluctuations. These complications pose significant challenges in achieving optimal management through pharmacological interventions (5, 6).

Hence, interventions such as deep brain stimulation (DBS) or the more novel method of magnetic resonance-guided focused ultrasound surgery (MRgFUS) are increasingly selected as approaches for patients resistant to medication or experience disabling motor complications. Numerous studies have shown that DBS and MRgFUS may be more effective than dopamine-related drugs in improving motor symptoms and QoL in PD (7–9).

Individuals who experience motor complications from drug therapy and undergo DBS often exhibit superior outcomes compared to those solely reliant on medication. Improvements include reduced motor symptoms, decreased dependence on dopaminergic medications, and enhanced self-assessed QoL (10, 11). Over the years, it has proven that DBS of the internal globus pallidus (GPi_DBS) and the subthalamic nucleus (STN_DBS) serves as an effective surgical procedure for managing motor fluctuations in PD patients (5, 12–15).

The academic literature widely accepts that GPi stimulation improves tremor, rigidity, and bradykinesia, whereas STN stimulation demonstrates comparable efficacy in symptom control while allowing for a reduction in dopaminergic therapy. In contrast, the ventral intermediate nucleus (VIM) holds a slight advantage in tremor control (7). To date, the use of DBS targeting both STN and GPi remains the leading surgical approach for managing PD.

Furthermore, the advent of MRgFUS, a novel incisionless technique capable of targeting the STN, GPi, or other brain regions, may advance its utilization (8, 16). The actions of MRgFUS in the brain are diverse, encompassing neuromodulation, opening of the blood–brain barrier, and thermal ablation of targeted tissues (17). In contrast to DBS, MRgFUS carries a minimal risk of hardware-related infection and hemorrhage. In recent years, some clinical studies have observed that VIM_MRgFUS can improve tremor-dominated Parkinson’s disease, while MRgFUS targeting STN and GPi can provide better performance for the motor symptoms (18).

Despite existing evidence, the safety and efficacy of MRgFUS remain limited, but the use of DBS in PD may offer valuable insights for clinicians in identifying potential targets for MRgFUS. Consequently, MRgFUS holds promise for greater adoption in clinical practice.

So far, clinical research has rarely compared the effectiveness of DBS targeting different brain regions with MRgFUS for PD. Unlike a pairwise meta-analysis, which compares two treatments, a network meta-analysis (NMA) evaluates the effectiveness of more than two treatments simultaneously. Previous research performed a network meta-analysis on the efficacy of DBS and MRgFUS in controlling PD-induced tremors, revealing a comparable potency in tremor reduction (7). Moreover, treatments such as GPi_DBS, GPi_MRgFUS, STN_DBS, and caudal zona incerta (cZi_DBS) showed noticeable improvements in motion-related symptoms compared to baseline (7).

However, this study did not compare these two surgical techniques directly with sole medical treatment (MT), nor did it focus on the aspect of quality of life (QoL). An analysis found that when it came to enhancing patient QoL in parkinsonism, both GPi_DBS and STN_DBS outperformed pharmacological therapy (19). Yet, there was no statistically significant difference between these DBS treatments, with the ranking probability showing that GPi_DBS was second to STN_DBS.

In the light of this background, we performed a NMA to indirectly compare the efficacy of DBS, MRgFUS and MT on motor performance and quality of life in PD patients. Subsequently, a comparative analysis was conducted to rank the efficacy of DBS and MRgFUS targeting various brain regions, along with medical treatment, in improving motor performance and quality of life.

## Methods

The current NMA adhered to the guidelines specified in the expanded checklist for preferred reporting items in systematic reviews and meta-analyses.

### Prospero registration number

PROSPERO CRD42024521903.

### Data sources and searches

To facilitate this meta-analysis, an extensive literature search was conducted, covering articles published from January 1998 to October 2023. Three prominent databases, namely PubMed, Embase, and Cochrane Library, were utilized for this purpose. The search included literature in multiple languages; however, only English-language publications was deemed appropriate for inclusion. The complete strategy is described in the Supplementary material.

### Inclusion criteria

1. Study subjects: individuals who have received a clinical diagnosis of PD.
2. Intervention: Patients with PD were divided into two groups: the intervention group received either DBS or MRgFUS, and the control group received medication treatment alone (MT). The specific therapeutic methods are as follows: STN_FUS, Gpi_FUS, VIM_FUS, STN_DBS, Gpi_DBS, STN-Gpi_DBS, STN-SNr_DBS, SNr_DBS, cZi_DBS, NBM_DBS, MT.
3. Outcomes: The studies employed the Unified Parkinson’s Disease Rating Scale, Part III (UPDRS-III or MDS-UPDRS-III), to assess motor symptoms, and evaluated quality of life using instruments such as the Parkinson’s Disease Questionnaire (PDQ-39/PDQ-8) and Sickness Impact Profile (SIP), to measure therapy effectiveness.

### Exclusion criteria

1. The exclusion criteria encompassed secondary parkinsonism, severe dementia, and significant concurrent depression.
2. If data extraction was not feasible or if the data lacked integrity.
3. Studies that were not clinical trials or those involving non-human subjects (such as mice or dogs), were excluded from the review.

### Evaluation of quality and information gathering

We used the Cochrane Collaboration’s tool to assess the quality of all trials, which consists of seven domains: generation of random sequences, concealment of allocations, blinding of personnel and outcome assessors, incomplete outcome data, selective reporting, and other biases. Two researchers from our team independently scrutinized the complete text of all suitable studies. In instances of discord, a third team researcher was involved in discussions to reach a final agreement. Based on the trials included, we gathered the subsequent data: the principal author’s identity, year of publication, demographic details, objectives, disease progression, UPDRS-III, and QoL scores.

### Outcome measures

The UPDRS and MDS-UPDRS (revised version) are widely used to assess functional status and motor symptoms in PD patients. Part III of both scales was utilized to evaluate motor function, with total scores ranging from 0 to 108 for the UPDRS-III and 0 to 132 for the MDS-UPDRS-III. The PDQ-39, its abbreviated version (PDQ-8), and the SIP are commonly used and important tools for assessing the QoL. Higher scores on these scales indicate greater severity of impairment.

### Statistical analysis

We used Stata Statistical Software, V.17 (StataCorp) for statistical analysis. Our approach involved conducting a frequentist meta-analysis, which does not require a prior distribution, thus avoiding subjective bias and simplifying implementation. To visualize each outcome, we used the ‘network plot’ command in Stata. The results of the NMA are presented as standardized mean differences (SMDs), which quantify the difference between two means on a unified scale, with 95% confidence intervals. The ability to assess the consistency assumption was limited because the networks did not include any closed loops. Using the Surface Under the Cumulative Ranking (SUCRA) method, we evaluated treatments, assigning each a score from 0 (least effective) to 100% (most effective) based on overall ranking. An investigation into the influence of the small sample size was performed by using funnel charts.

## Results

**Figure 1:**
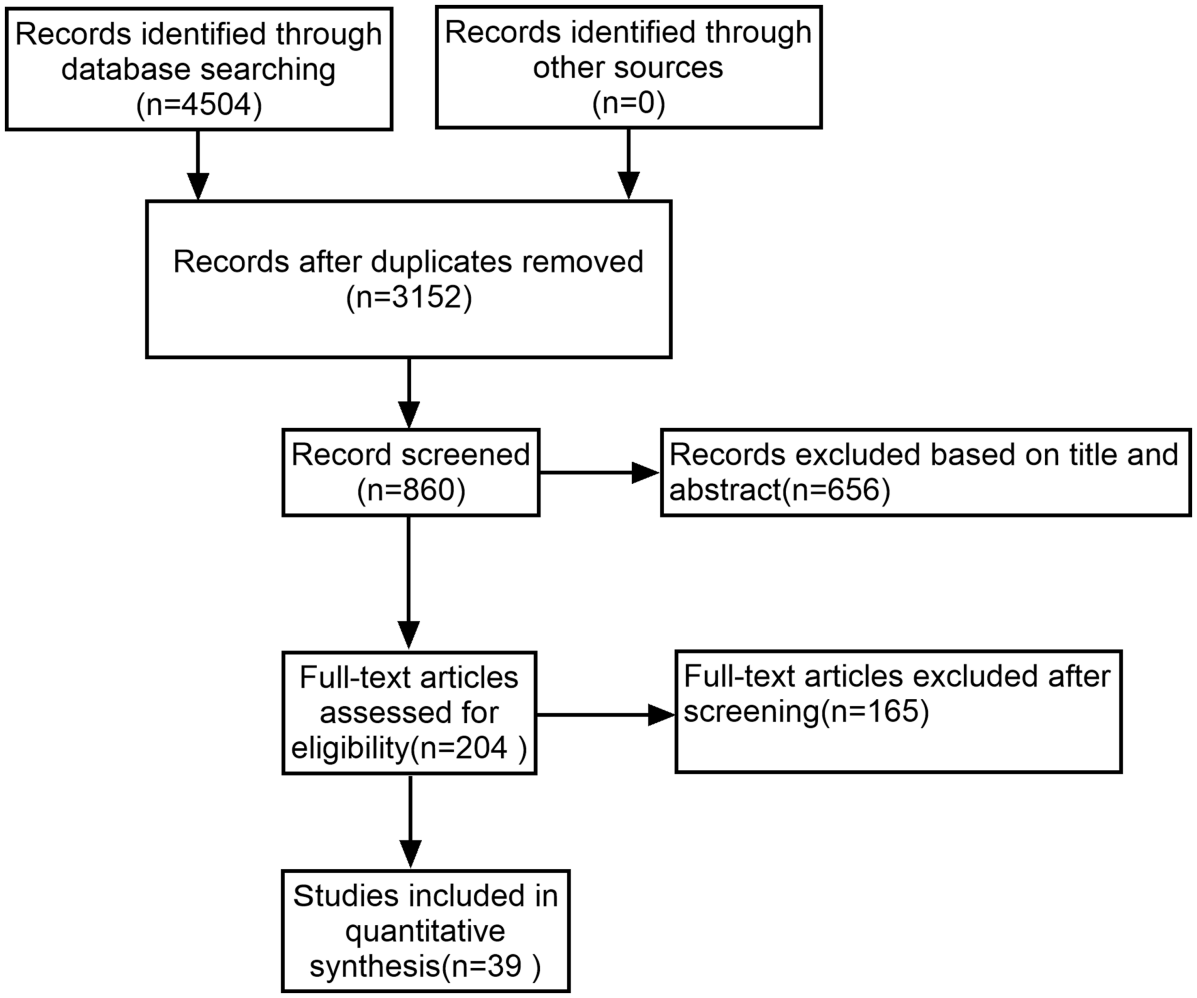
Flow diagram.

A thorough literature search initially identified 4,506 studies, from which 1,354 duplicates were removed. After applying inclusion and exclusion criteria, 39 of the remaining 204 studies were selected for inclusion in this NMA, encompassing 3,002 patients with PD (Figure 1).

### Basic characteristics

**Figure 2:**
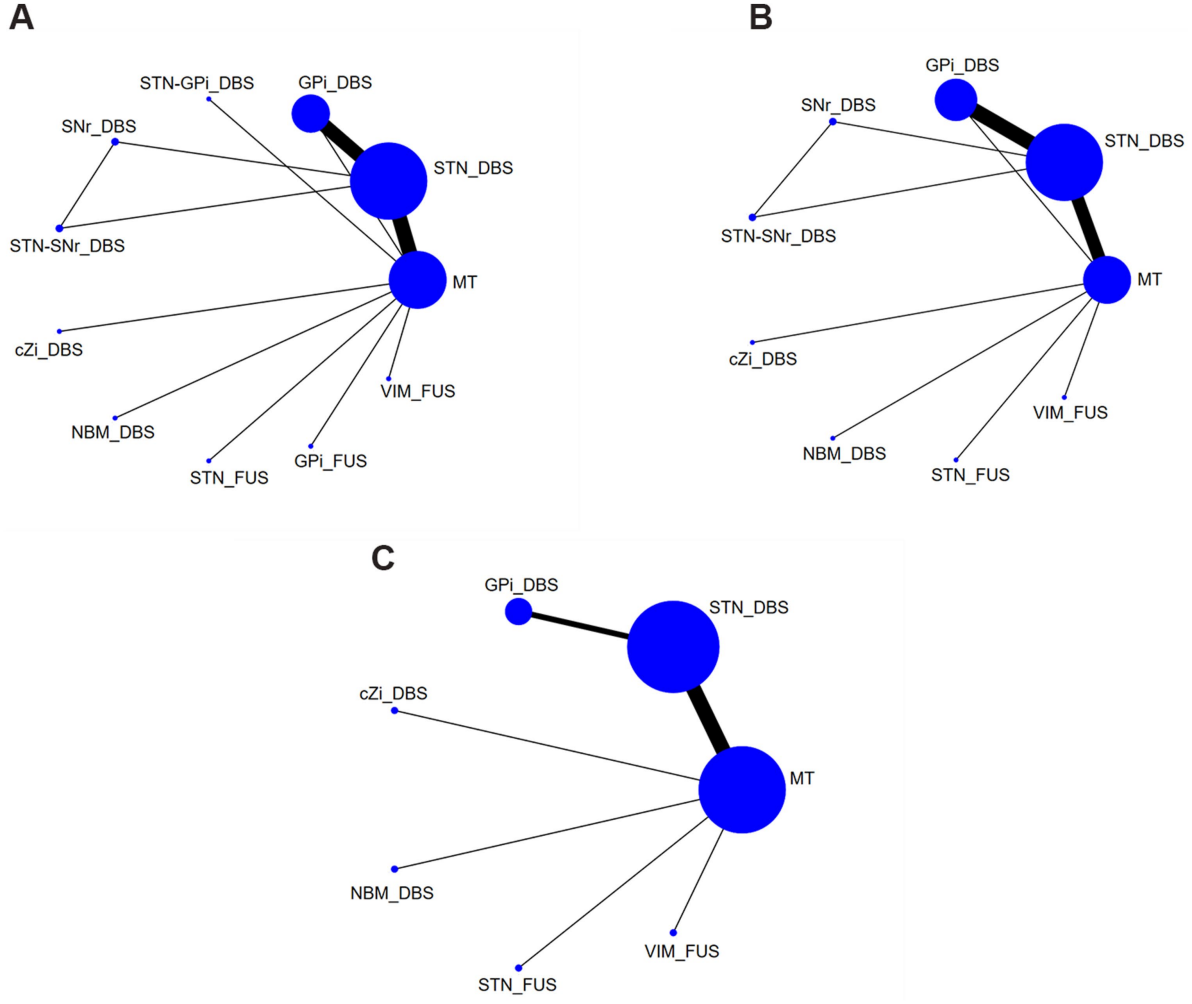
Network plots of each evaluation. **(A)** UPDRS-III Total score (off-phase); **(B)** UPDRS-III Total score (on-phase); **(C)** QoL. DBS, deep brain stimulation; FUS, focused ultrasound surgery; STN, subthalamic nucleus; Gpi, internal globus pallidus; cZi, caudal zona incerta; NBM, nucleus basalis of Meynert; SNr, substantia nigra pars reticulata; VIM, ventral intermediate nucleus; MT, medication treatment.

Table 1 summarizes baseline characteristics of the participants in the included trials. Figures 2A–C presents the network plots for each treatment target of DBS, FUS or MT.

**Table 1:** Comparative characteristics of distinct targets.

Number	Author & Year	Treatment	Surgical modus	Sample size, n	Age, years	Male/female, n	Disease duration, years	LEDD at base line, mg	Follow-up periods, months	Outcomes
1	Krishna 2023 (8)	GPi_FUS	uni	65	64.20 ± 9.60	43/25	NA	1051.60 ± 473.80	3	MDS–UPDRS–III
		MT*	–	22	63.30 ± 9.20	14/10	NA	1044.70 ± 660.60		
2	Andreasi 2022 (38)	VIM_FUS	NA	10	62.30 (60.20; 72.30)	8/2	3.80 (2.40; 4.50)	472.50 (300.00; 650.00)	6	MDS–UPDRS–III
		MT	–	20	62.87 (59.50; 72.10)	16/4	3.20 (2.80; 4.10)	400.00 (285.00; 525.00)		
3	Weiss 2022 (9)	STN_DBS	NA	84	52.40 ± 7.00	66/18	7.20 ± 2.70	942.30 ± 47.00	24	UPDRS–III, PDQ39
		MT	–	89	52.30 ± 5.80	59/30	7.60 ± 2.60	980.30 ± 46.00		
4	Zeng 2022 (39)	STN_DBS	uni	8	66.13 ± 6.71	5/3	10.13 ± 7.85	616.97 ± 276.04	24 ~ 36	UPDRS–III
		GPi_DBS	uni	8	66.13 ± 6.71	5/3	10.13 ± 7.85	616.97 ± 276.04		
5	Jost 2021 (40)	STN_DBS	bi	40	62.20 ± 8.60	25/15	9.70 ± 4.70	1066.00 ± 468.20	36	PDQ8
		MT	–	40	63.80 ± 10.40	27/13	8.30 ± 4.90	885.20 ± 355.30		
6	Dafsari 2020 (41)	STN_DBS	bi	28	58.50 ± 12.40	19/11	10.40 ± 5.60	1164.10 ± 449.20	6	PDQ8
		GPi_DBS	bi	18	58.10 ± 9.10	11/7	11.00 ± 4.00	1166.20 ± 563.20		
7	Hacker 2020 (42)	STN_DBS	bi	14	NA	NA	NA	526.7 ± 313.0	24	PDQ39
		MT	–	14	NA	NA	NA	705.2 ± 377.1		
8	Li 2020 (43)	STN_DBS	bi	16	60.25 ± 5.56	8/8	10.38 ± 4.33	1225.63 ± 714.81	6	MDS–UPDRS–III, PDQ39
		MT	–	20	57.88 ± 6.98	8/12	12.85 ± 4.25	1200.80 ± 714.81		
9	Martínez-Fernández 2020 (44)	STN_FUS	uni	27	56.60 ± 9.30	16/11	5.60 ± 2.50	729.70 ± 328.30	4	MDS–UPDRS–III, PDQ39
		MT*	–	13	58.10 ± 8.80	10/3	7.30 ± 3.80	881.70 ± 407.90		
10	Martinez-Martin 2020 (45)	STN_DBS	NA	120	NA	NA	NA	NA	24	PDQ39
		MT	–	123	NA	NA	NA	NA		
11	Vitek 2020 (46)	STN_DBS	bi	121	60.70 ± 7.90	90/31	10.00 ± 3.60	1252.20 ± 843.00	3	UPDRS–III, PDQ39
		MT*	–	39	57.50 ± 7.70	26/12	10.20 ± 3.60	1456.00 ± 1004.00		
12	Zhang 2020 (47)	STN–GPi_DBS	bi	8	67.38 ± 4.81	7/1	10.13 ± 4.36	777.34 ± 264.11	6	UPDRS–III
		MT	–	8	67.38 ± 4.81	7/1	10.13 ± 4.36	777.34 ± 264.11		
13	Valldeoriola 2019 (48)	STN_DBS	bi	6	59.10[43–70.00]	5/1	16.1.[10.00–20.00]	1250.00 ± 427.00	3	UPDRS–III
		SNr_DBS	bi	6	59.10[43–70.00]	5/1	16.1.[10.00–20.00]	1250.00 ± 427.00		
		STN–SNr_DBS	bi	6	59.10[43–70.00]	5/1	16.1.[10.00–20.00]	1250.00 ± 427.00		
14	Blomstedt 2018 (49)	cZi_DBS	bi	9	57.00 ± 11.40	7/2	6.40 ± 3.00	1376.00 ± 883.00	6	UPDRS–III, PDQ39
		MT	–	10	60.90 ± 9.20	8/2	10.30 ± 5.60	1043.00 ± 516.00		
15	Gratwicke 2018 (50)	NBM_DBS	bi	6	65.20 ± 10.70	6/0	12.70 ± 2.30	646.90 ± 204.70	1.5	MDS–UPDRS–III, PDQ39
		MT*	–	6	65.20 ± 10.70	6/0	12.70 ± 2.30	646.90 ± 204.70		
16	Bond 2017 (51)	VIM_FUS	uni	20	68.1 (63.70;73.30)	19/1	5.90 (3.40;9.20)	751.00 (450.00;950.00)	3	UPDRS–III, PDQ39
		MT*	–	7	62.40 (50.20;76.20)	7/0	6.70 (5.40;8.10)	640.00 (550.00;1250.00)		
17	Hacker 2015 (52)	STN_DBS	bi	9	60.00 ± 5.60	9/0	2.70 ± 1.30	475.70 ± 323.10	12	UPDRS–III, PDQ39
		MT	–	11	60.00 ± 7.50	9/2	2.10 ± 0.90	479.30 ± 242.70		
18	St George 2015 (53)	STN_DBS	bi	11	62.00 ± 5.70	9/2	13.30 ± 5.00	1349.00 ± 668.00	6	UPDRS–III
		GPi_DBS	bi	10	62.80 ± 8.20	9/1	15.40 ± 8.70	1412.00 ± 887.00		
		MT	–	8	60.00 ± 8.50	7/1	12.10 ± 6.00	1253.00 ± 47.00		
19	Charles 2014 (54)	STN_DBS	bi	15	60.00 ± 6.80	14/1	2.20 ± 1.40	417.20 ± 306.60	24	UPDRS–III, PDQ39
		MT	–	14	60.00 ± 7.00	NA	2.10 ± 1.10	494.00 ± 208.70		
20	Okun 2014 (55)	STN_DBS	bi	16	58.00 ± 10.70	13/3	12.10 ± 4.50	805.40 ± 434.70	4	UPDRS–III
		GPi_DBS	bi	14	58.00 ± 10.70	8/6	11.50 ± 3.30	1037.10 ± 647.80		
21	Schuepbach 2013 (10)	STN_DBS	bi	124	52.90 ± 6.60	94/30	7.30 ± 3.10	918.80 ± 412.50	24	UPDRS–III, PDQ39
		MT	–	127	52.20 ± 6.10	85/42	7.70 ± 2.70	966.90 ± 416.50		
22	Chang 2012 (56)	STN_DBS	bi	31	58.32 ± 4.18	20/11	NA	814.31 ± 195.49	7	PDQ39
		MT	–	31	57.83 ± 4.23	20/11	NA	826.86 ± 218.05		
23	Okun 2012 (57)	STN_DBS	bi	100	60.60 ± 8.30	NA	12.10 ± 4.90	1311.00 ± 615.00	3	UPDRS–III
		MT	–	35	59.50 ± 8.20	21/14	11.70 ± 4.10	1459.00 ± 991.00		
24	Rocchi 2012 (58)	STN_DBS	bi	15	61.40 ± 5.50	11/4	11.90 ± 4.80	1313.10 ± 670.20	6	UPDRS–III
		GPi_DBS	bi	14	61.10 ± 8.40	13/1	12.90 ± 10.17	1305.90 ± 667.40		
25	Weaver 2012 (20)	STN_DBS	NA	67	60.70 ± 8.90	NA	NA	1270.00 ± 570.00	6	UPDRS–III, PDQ39
		GPi_DBS	NA	83	60.40 ± 8.30	NA	NA	1365.00 ± 543.00		
26	Robertson 2011 (59)	STN_DBS	bi	14	63.80 ± 6.30	13/1	16.80 ± 6.20	1289.00 ± 652.00	6	UPDRS–III
		GPi_DBS	bi	13	65.50 ± 8.60	12/1	15.10 ± 10.20	1306.00 ± 667.00		
27	Smeding 2011 (60)	STN_DBS	bi	99	57.90 ± 8.10	58/41	13.70 ± 6.10	899.30 ± 498.00	6	PDQ39
		MT	–	36	63.00 ± 9.10	21/15	10.40 ± 4.60	629.60 ± 304.90		
28	Follett 2010 (61)	STN_DBS	bi	147	61.90 ± 8.70	116/31	NA	1118.00 ± 562.00	24	UPDRS–III, PDQ39
		GPi_DBS	bi	152	61.80 ± 8.70	133/19	NA	1361.00 ± 545.00		
29	Moro 2010 (62)	STN_DBS	bi	31	59.30 ± 9.47	NA	15.30 ± 6.51	1709.30 ± 986.80	3	UPDRS–III
		GPi_DBS	bi	15	56.00 ± 8.40	NA	15.10 ± 6.00	1417.80 ± 612.00		
30	Montel 2009 (63)	STN_DBS	NA	40	56.00 ± 9.20	22/18	11.90 ± 5.00	975.00 ± 443.80	12	UPDRS–III
		MT	–	40	55.80 ± 9.30	22/18	11.00 ± 4.40	1065.00 ± 576.80		
31	Volkmann 2009 (64)	STN_DBS	bi	45	58.50 ± 9.80	22/23	15.30 ± 6.30	NA	6	UPDRS–III, SIP
		GPi_DBS	bi	20	55.80 ± 9.40	7/13	15.40 ± 6.20	NA		
32	Zahodne 2009 (65)	STN_DBS	uni	20	61.30 ± 9.00	14/6	13.57 ± 3.88	935.90 ± 374.00	6	UPDRS–III, PDQ39
		GPi_DBS	uni	22	61.30 ± 5.50	16/6	12.36 ± 3.58	1199.80 ± 576.90		
33	Zangaglia 2009 (66)	STN_DBS	bi	32	58.84 ± 7.70	18/14	11.84 ± 5.07	617.19 ± 303.57	36	UPDRS–III
		MT	–	33	62.52 ± 6.82	20/13	9.97 ± 4.86	647.73 ± 243.78		
34	Witt 2008 (67)	STN_DBS	bi	60	60.20 ± 7.90	36/24	13.80 ± 6.30	1203.00 ± 535.00	6	UPDRS–III
		MT	–	63	59.40 ± 7.50	41/22	14.00 ± 6.10	1142.00 ± 463.00		
35	Deuschl 2006 (68)	STN_DBS	bi	71	60.50 ± 7.40	NA	NA	1176.00 ± 517.00	6	UPDRS–III, PDQ39
		MT	–	73	60.80 ± 7.80	NA	NA	1175.00 ± 461.00		
36	Anderson 2005 (69)	STN_DBS	bi	10	61.00 ± 9.00	NA	15.60 ± 5.00	NA	12	UPDRS–III
		GPi_DBS	bi	10	54.00 ± 12.00	NA	10.30 ± 2.00	NA		
37	Capecci 2005 (70)	STN_DBS	bi	23	59.50 (7.50)	12/11	12.80 (4.20)	987.87 (427.00)	12	UPDRS–III
		MT	–	16	62.20 (6.50)	6/10	10.30 (4.20)	961.19 (474.00)		
38	Just 2002 (71)	STN_DBS	bi	11	59.80 (6.80)	8/3	14.00 (6.00)	NA	6	PDQ39
		MT	–	13	61.40 (5.70)	7/6	16.00 (6.00)	NA		
39	Obeso 2001 (14)	STN_DBS	bi	91	59.00 ± 9.60	NA	NA	1218.80 ± 575.00	6	UPDRS–III
		GPi_DBS	bi	36	55.70 ± 9.80	NA	NA	1090.90 ± 543.00		

Data are expressed as number, mean±SD, median (interquartile range), mean [range]. *: medical treatment & sham procedure or off stimulation. NA, not available. DBS, deep brain stimulation; FUS, focused ultrasound surgery; STN, subthalamic nucleus; Gpi, internal globus pallidus; cZi, caudal zona incerta; NBM, nucleus basalis of Meynert; SNr, substantia nigra pars reticulata; VIM, ventral intermediate nucleus; MT, medication treatment; uni, unilateral; bi, bilateral; UPDRS-III, Unified Parkinson’s Disease Rating Scale, Part III; PDQ, Parkinson’s Disease Questionnaire; SIP, Sickness Impact Profile.

### Risk of bias

**Figure 3:**
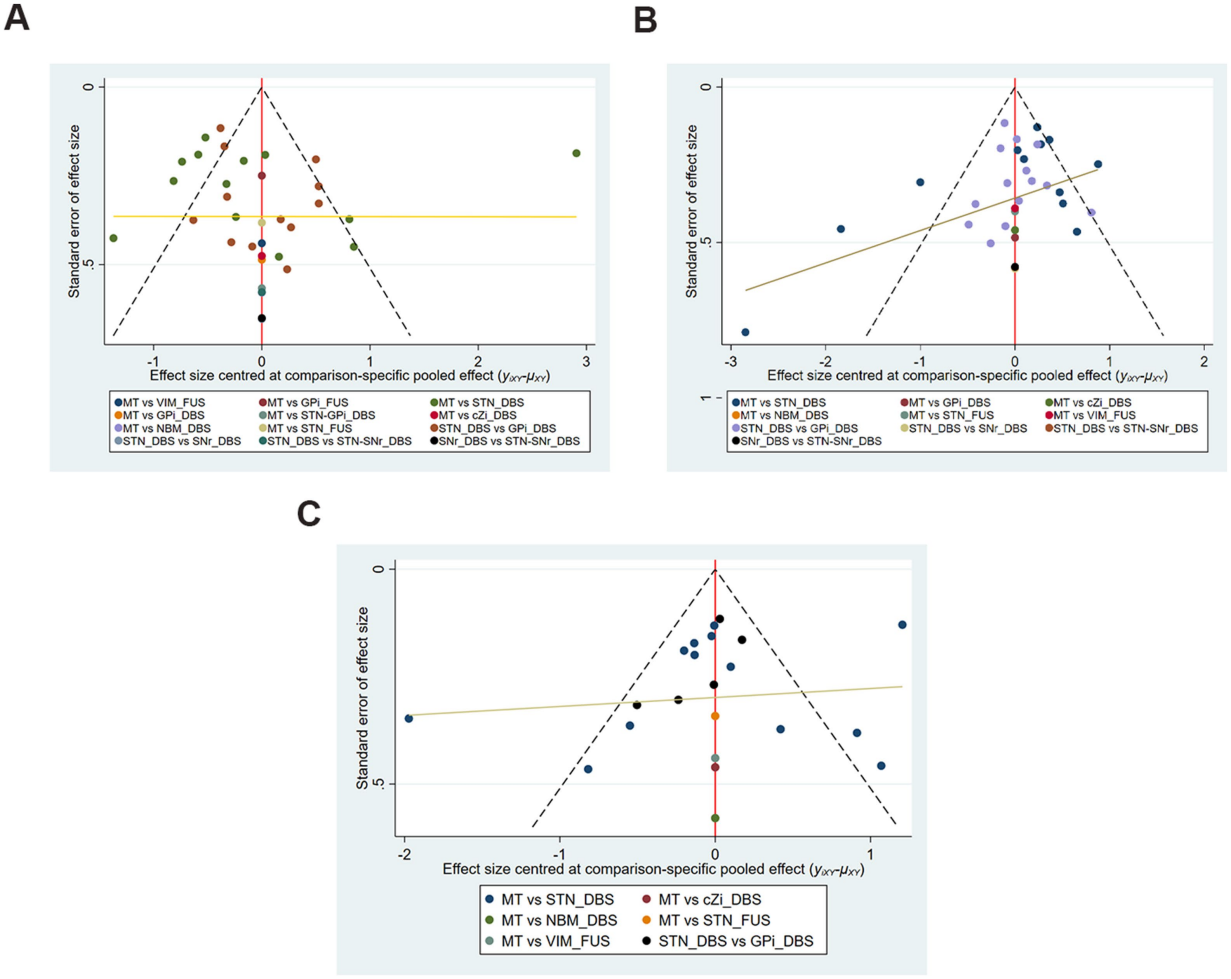
Funnel plots of each evaluation. **(A)** UPDRS-III Total score (off-phase); **(B)** UPDRS-III Total score (on-phase); **(C)** QoL.

**Figure 4:**
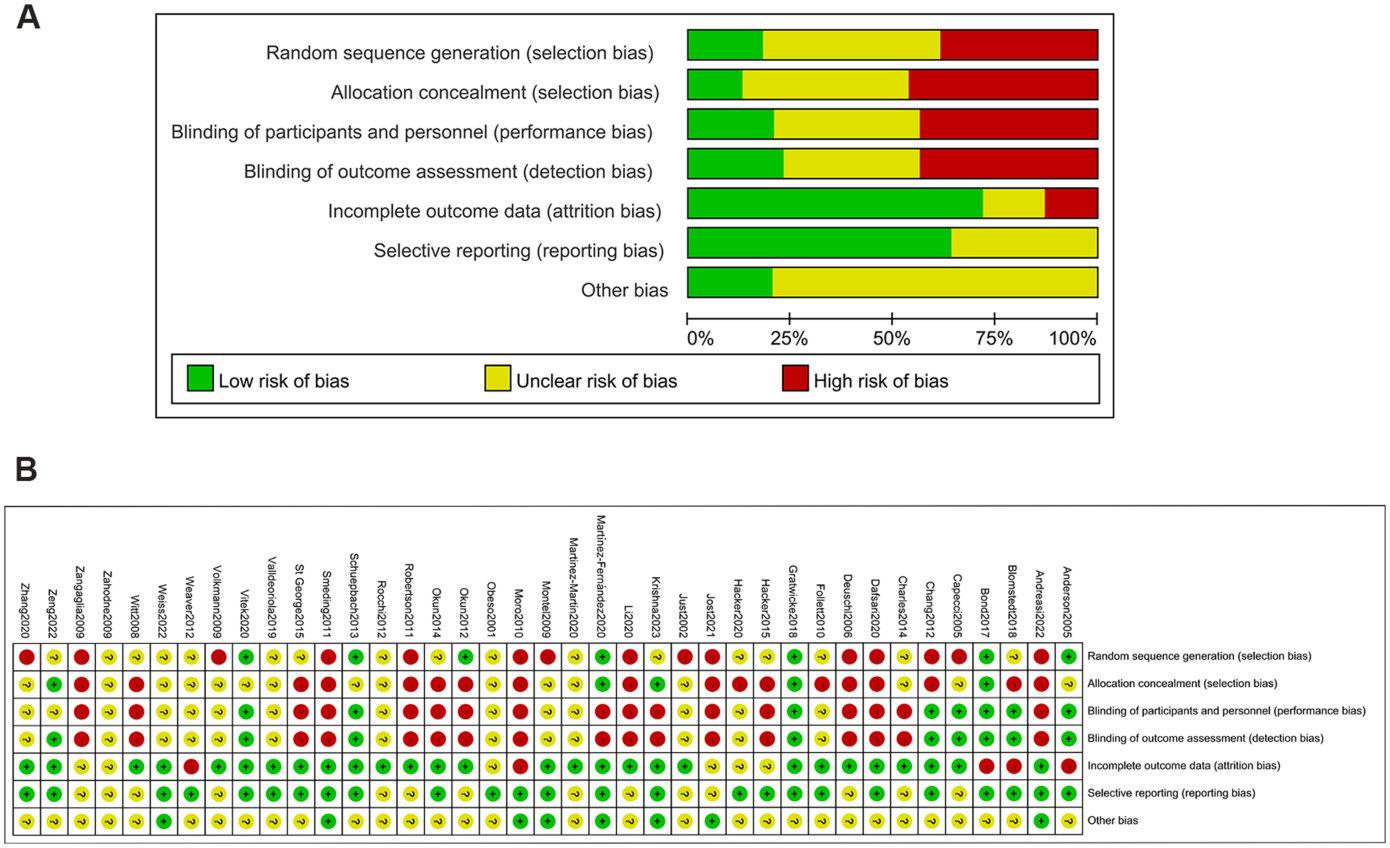
Risk of bias assessment. **(A)** Risk of bias graph; **(B)** Risk of bias summary.

Figures 3A–C shows no significant publication bias; the effect of small sample effect is minimal. The risk of bias for the included trials is displayed in Figures 4A,B.

### UPDRS-III total score (off-phase)

The analysis included a total of 31 studies (29 two-arm and 2 three-arm) examining the UPDRS III scores in the off-phase. These studies involved 11 treatment modalities, encompassing a total of 2,350 patients: STN_DBS, GPi_DBS, STN-GPi_DBS, substantia nigra pars reticulata_DBS (SNr_DBS), STN-SNr_DBS, cZi_DBS, nucleus basalis of Meynert_DBS (NBM_DBS), STN_FUS, GPi_FUS, VIM_FUS, and MT.

**Figure 5:**
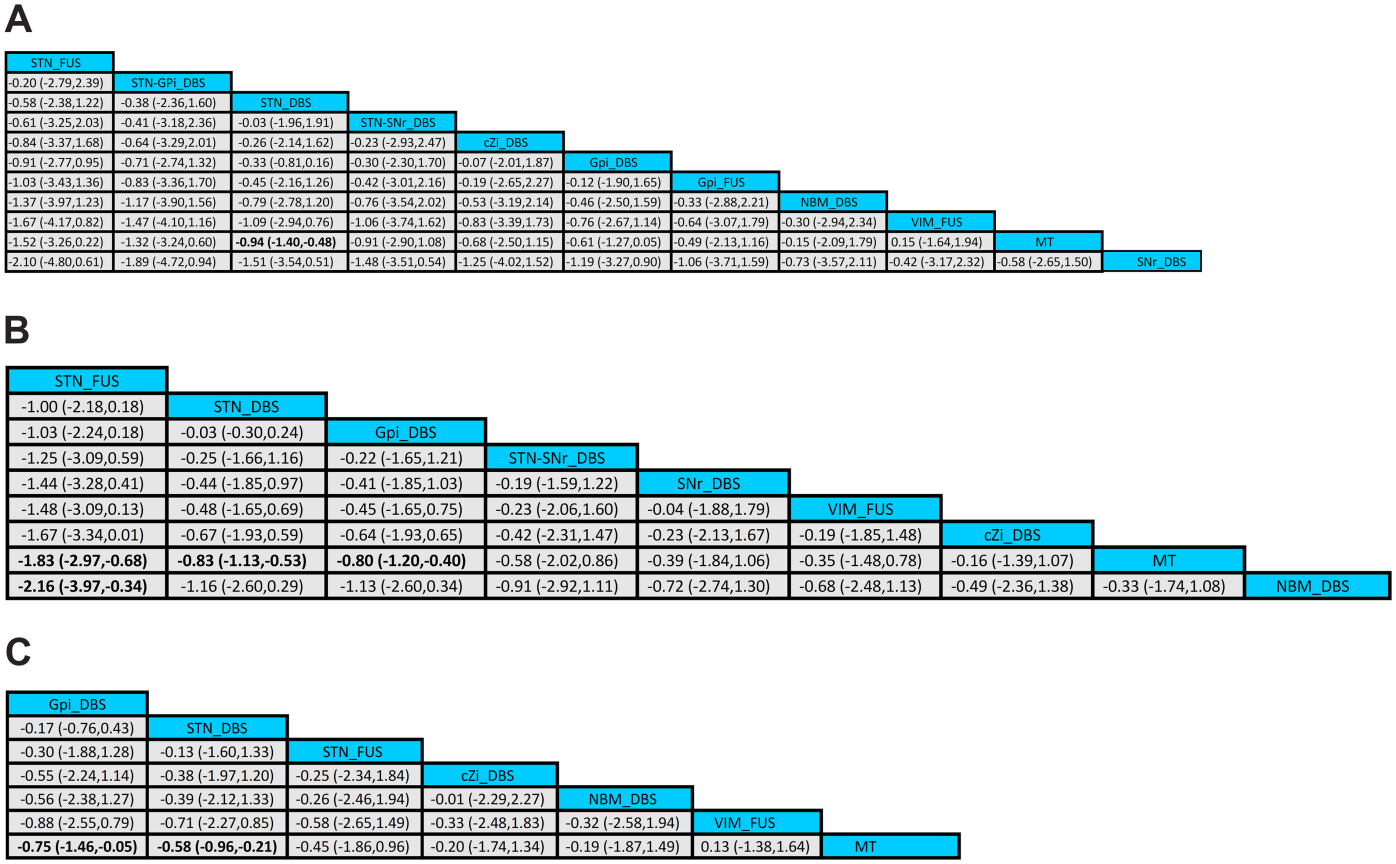
League tables of the NMA outcome. **(A)** UPDRS-III (off-phase); **(B)** UPDRS-III (on-phase); **(C)** QoL.

**Figure 6:**
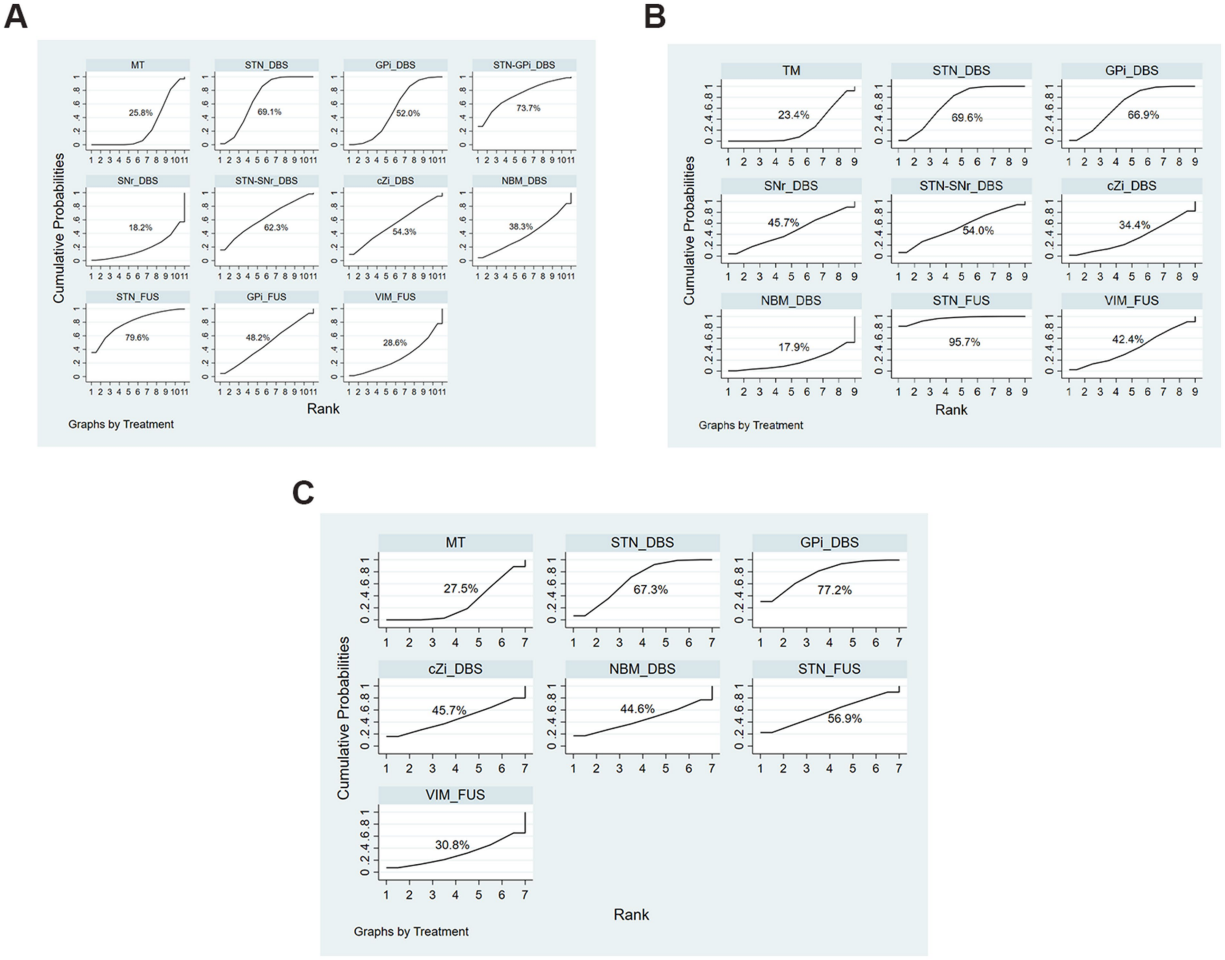
SUCRA Plots of each evaluation. **(A)** UPDRS-III Total score (off-phase); **(B)** UPDRS-III Total score (on-phase); **(C)** QoL.

In comparison, treatment with STN_DBS resulted in significant improvements in UPDRS-III scores compared to MT (SMD, −0.94; 95% CI, −1.40 to −0.48) in the off-phase (Figure 5A). According to the SUCRA plot (Figure 6A), the top three treatments were as follows: STN_FUS (79.6%) ranked first, followed by STN-GPi_DBS (73.7%) in second place, and STN_DBS (69.1%) in third, while SNr_DBS (18.2%) ranked last.

### UPDRS-III total score (on-phase)

The comparison of UPDRS-III in the on-phase incorporated 30 studies (28 two-arm and 2 three-arm) and 9 treatments used in 2184 patients, including STN_DBS, GPi_DBS, SNr_DBS, STN-SNr_DBS, cZi_DBS, NBM_DBS, STN_FUS, VIM_FUS, and MT.

In the on-phase, significant improvements in UPDRS-III scores were observed with STN_DBS (SMD, −0.83; 95% CI, −1.13 to −0.53), GPi_DBS (SMD, −0.80; 95% CI, −1.20 to −0.40), and STN_FUS (SMD, −1.83; 95% CI, −2.97 to −0.68) compared to MT (Figure 5B). According to the SUCRA plot (Figure 6B), the top three techniques were STN_FUS (95.7%) in first place, followed by STN_DBS (69.6%), GPi_DBS (66.9%) in third place, while NBM_DBS (17.9%) was in the last position.

### Quality of life

The QoL assessment included 22 studies, involving 2085 patients, and compared seven two-arm treatment strategies: STN_DBS, GPi_DBS, cZi_DBS, NBM_DBS, STN_FUS, VIM_FUS, and MT.

Among all treatments, significant improvements in QoL were observed with STN_DBS (SMD, −0.75; 95% CI, −1.46 to −0.05) and GPi_DBS (SMD, −0.58; 95%CI, −0.96 to −0.21) compared to MT (Figure 5C). According to the SUCRA plot (Figure 6C), the top three interventions were: GPi_DBS (77.2%) in first place, followed by STN_DBS (67.3%) in second place, and STN_FUS (56.9%) in third place, with MT (27.5%) ranking last.

### Adverse event

The included studies generally reported nucleus-related, PD-related, Procedure or device-related, dopaminergic therapy-related or other adverse events. Certain studies differentiate between severe and non-severe adverse events, while others omit the inclusion of adverse effects altogether. A range of adverse events noted in the included studies is detailed in the Supplementary material.

## Discussion

We analyzed 39 clinical trials involving 3,002 PD patients and compared different targets of DBS and MRgFUS. This study found that STN_DBS significantly enhanced motor symptoms in both the off-phase and on-phase compared with MT. Additionally, both GPi_DBS and STN_FUS demonstrated significant improvement in the on-phase.

We utilized the Surface Under the Cumulative Ranking curve (SUCRA) to assess the probability of each treatment being the most effective option. SUCRA values range from 0 to 100%, with a value closer to 100% indicating a higher likelihood of being the most effective intervention.

Although not statistically significant in the off-phase, STN_FUS consistently ranked the top position in the SUCRA ranking in both the on-phase and off-phase, hinting at potential advancements in motor symptoms. Additionally, it is important to note that STN_DBS and GPi_DBS significantly impact QoL, with STN_DBS ranking first, GPi_DBS ranking second, and STN_FUS ranking third in effectiveness.

Numerous clinical studies have substantiated the significant contribution of STN_DBS and GPi_DBS in ameliorating motor behavior compared to dopaminergic medications alone (5, 9, 10, 20). A network meta-analysis, comparing various targets of DBS, indicated that both STN_DBS and GPi_DBS exhibit potential for enhancing both motor and non-motor symptoms (21). It is highly plausible that STN_DBS yields equivalent outcomes to GPi_DBS in the treatment of motor performance and QoL (22, 23). However, our research could not clarify the differential impacts of STN_DBS and GPi_DBS for their effectiveness in augmenting exercise performance and quality of life.

There is a currently prevailing belief that STN_DBS is more efficient than GPi_DBS in reducing reliance on dopaminergic medications, although it has a higher propensity to impair cognitive function. This potential effect may arise because the lesion locations affecting cognitive function and the STN_DBS target area is part of the same brain network. Consequently, connectivity between STN_DBS sites and cognition-related region was significantly associated with cognitive decline following DBS (24). Meanwhile, blocking dopamine terminals in the STN boosts its activity, showing dopamine’s direct influence on the STN (25).

While a definitive cure for PD continues to be elusive, there exist effective treatments to manage the symptoms. DBS is one such treatment that has consistently proven its effectiveness. Thalamic DBS is optimal for handling tremors. Pallidum DBS has been shown to be excellent for rigidity and dyskinesias. STN DBS can manage a range of symptoms and decrease the requirement for medications, thereby earning recognition as a favored DBS focus area (5).

Despite its benefits, DBS carries the risk of certain complications, which discourages many patients from opting for the invasive procedure (26, 27). Adverse reactions of DBS include dysarthria, changes in mood or cognition impairment, implant infection, and other adverse outcomes (10). A small number of patients may experience serious adverse effects related to the device (28).

Nonetheless, our study focused more on STN_FUS. MRgFUS generates extracorporeal ultrasound to deliver ultrasonic energy precisely to specific brain regions through the skull, allowing for incision-free lesion treatment and real-time monitoring (29). Different points of focus have been employed for MRgFUS in the handling of PD; these include areas like the ventral lobe of VIM, STN, GPi, along with pallidothalamic tractotomy (PTT) (17). In comparison to DBS, MRgFUS does not necessitate implantation of a device and presents a minimal risk of hemorrhage and infection (30, 31).

The case series by Schlesinger et al. pointed out that VIM_FUS can simultaneously improve tremor severity, UPDRS-III and PDQ-39 scores in PD individuals (32), while Moosa et al. summarized previous studies in a review and concluded that MRgFUS of the VIM, STN, and GPi all can improve patients’ motor symptoms and produce fewer adverse reactions than DBS (29).

Parkinsonian symptoms occur when output from the GPi or SNr is excessively inhibited, affecting thalamic cortical projections, which is induced by an increase in STN excitatory activity (33). Therefore, MRgFUS is theoretically feasible and effective for STN and GPi targets.

Because we only included one study on the effect of GPi_FUS on motor symptoms, our results on FUS are limited; this study has only found that STN_FUS may improve motor symptoms and QoL. Due to its anatomical position relative to the STN or VIM, targeting the GPi may require steeper angles of the ultrasound beam, which could reduce energy transfer efficiency (17). Hence these potential problems limit the current results. Additionally, the outcomes of our VIM_FUS procedure did not yield favorable results, possibly due to the anatomical challenges associated with targeting this region. In contrast to our findings, Schlesinger and colleagues reported notable improvements in motor abilities and Quality of Life in a group of seven patients suffering from PD, who had undergone unilateral VIM-MRgFUS treatment for managing tremors (32).

The STN_FUS improves both dyskinesia and QoL, which aligns with the conclusions of many advanced studies (18, 34, 35). Regrettably, only one study concerning STN_FUS was included in our analysis. In addition, we failed to find that GPi_FUS and VIM_FUS have a meaningful effect on motor function and QoL, which is contrary to the conclusions of other studies (36, 37).

Given the limited research on MRgFUS and smaller sample sizes, and lack of in-depth follow-up period, interpreting the results requires caution. Besides, MRgFUS treatment involves creating a lesion in the target region, precluding postoperative adjustments. In conclusion, despite certain drawbacks, MRgFUS represents a promising, less invasive alternative for treating PD, with the potential to offer benefits comparable to those of DBS.

### Limitations

Certain limitations are inevitable in this study. Currently, RCT studies on FUS are very rare. Our study includes only 27 patients targeting STN and 65 patients targeting GPi, hence the conclusions drawn from this may not be highly reliable. However, future RCT studies are expected to increase, and this new surgical technique sure will bring about new hope. We did not conduct rigorous subgroup analysis based on follow-up times, which could potentially limit comparisons of different outcomes. In addition, we did not perform statistical analysis on adverse reactions to surgery. Future studies should consider these variables.

In this study, we employed a frequentist NMA to evaluate the effects of different treatments on Parkinson’s disease patients. This approach allows for effective comparisons between treatments, providing interpretable effect sizes (e.g., SMD) and confidence intervals. However, it has limitations regarding sample heterogeneity and missing data. Although most studies included were of high quality, caution is warranted due to potential publication bias from lower-quality studies. Additionally, NMA relies on indirect comparisons from existing literature, lacking direct support from randomized controlled trials, which necessitates careful interpretation of treatment effects, particularly regarding their applicability to diverse patient populations.

## Conclusion

Surgical interventions such as STN_DBS, GPi_DBS, and STN_FUS have exhibited efficacy in ameliorating motor symptoms, alongside enhancing quality of life in parkinsonism. Moreover, indirect evidence from our study indicates that STN-FUS is not inferior to STN-DBS in both aspects for PD. Therefore, STN-FUS may serve as a second alternative with comparable efficacy to STN-DBS in the management of PD. In conclusion, based on the assessment of motor function improvements and quality of life, we provide recommendations for surgical treatment options. For motor symptoms in the off-phase, STN-DBS is the preferred approach. In the on-phase, STN-DBS, GPi-DBS and STN_FUS, are considered viable options. Regarding improvements in quality of life, STN-DBS and GPi-DBS are the preferred treatments. Taking all factors into account, STN-DBS is ultimately recommended as the optimal surgical intervention.

## References

[1] Postuma RB Berg D Stern M Poewe W Olanow CW Oertel W. MDS clinical diagnostic criteria for Parkinson’s disease. Mov Disord. (2015) 30:1591–601. doi: 10.1002/mds.26424, PMID: 26474316

[2] Belarbi K Cuvelier E Bonte M-A Desplanque M Gressier B Devos D. Glycosphingolipids and neuroinflammation in Parkinson’s disease. Mol Neurodegener. (2020) 15:59. doi: 10.1186/s13024-020-00408-1, PMID: 33069254 PMC7568394

[3] Dorsey ER Bloem BR. The Parkinson Pandemic-A Call to Action. JAMA Neurol. (2018) 75:9–10. doi: 10.1001/jamaneurol.2017.3299, PMID: 29131880

[4] Mao Q Qin WZ Zhang A Ye N. Recent advances in dopaminergic strategies for the treatment of Parkinson's disease. Acta Pharmacol Sin. (2020) 41:471–82. doi: 10.1038/s41401-020-0365-y, PMID: 32112042 PMC7471472

[5] Hariz M Blomstedt P. Deep brain stimulation for Parkinson's disease. J Intern Med. (2022) 292:764–78. doi: 10.1111/joim.13541, PMID: 35798568 PMC9796446

[6] Dong-Chen X Yong C Yang X Chen-Yu S Li-Hua P. Signaling pathways in Parkinson's disease: molecular mechanisms and therapeutic interventions. Signal Transduct Target Ther. (2023) 8:73. doi: 10.1038/s41392-023-01353-3, PMID: 36810524 PMC9944326

[7] Lin F Wu D Yu J Weng H Chen L Meng F. Comparison of efficacy of deep brain stimulation and focused ultrasound in parkinsonian tremor: a systematic review and network meta-analysis. J Neurol Neurosurg Psychiatry. (2021) 92:434–43. doi: 10.1136/jnnp-2020-323656, PMID: 33461975

[8] Krishna V Fishman PS Eisenberg HM Kaplitt M Baltuch G Chang JW. Trial of Globus Pallidus Focused Ultrasound Ablation in Parkinson's Disease. N Engl J Med. (2023) 388:683–93. doi: 10.1056/NEJMoa2202721, PMID: 36812432

[9] Weiss D Landoulsi Z May P Sharma M Schüpbach M You H. Genetic stratification of motor and QoL outcomes in Parkinson's disease in the EARLYSTIM study. Parkinsonism Relat Disord. (2022) 103:169–74. doi: 10.1016/j.parkreldis.2022.08.025, PMID: 36117018

[10] Schuepbach WM Rau J Knudsen K Volkmann J Krack P Timmermann L. Neurostimulation for Parkinson's disease with early motor complications. N Engl J Med. (2013) 368:610–22. doi: 10.1056/NEJMoa1205158, PMID: 23406026

[11] Lambert C Zrinzo L Nagy Z Lutti A Hariz M Foltynie T. Confirmation of functional zones within the human subthalamic nucleus: patterns of connectivity and sub-parcellation using diffusion weighted imaging. Neuroimage. (2012) 60:83–94. doi: 10.1016/j.neuroimage.2011.11.082, PMID: 22173294 PMC3315017

[12] Siegfried J Lippitz B. Bilateral chronic electrostimulation of ventroposterolateral pallidum: a new therapeutic approach for alleviating all parkinsonian symptoms. Neurosurgery. (1994) 35:1126–1130; discussion 1129-1130. doi: 10.1227/00006123-199412000-00016 7885558

[13] Bergman H Wichmann T DeLong MR. Reversal of experimental parkinsonism by lesions of the subthalamic nucleus. Science. (1990) 249:1436–8. doi: 10.1126/science.2402638, PMID: 2402638

[14] Obeso JA Olanow CW Rodriguez-Oroz MC Krack P Kumar R Lang AE. Deep-brain stimulation of the subthalamic nucleus or the pars interna of the globus pallidus in Parkinson's disease. N Engl J Med. (2001) 345:956–63. doi: 10.1056/NEJMoa000827, PMID: 11575287

[15] Harmsen IE Wolff Fernandes F Krauss JK Lozano AM. Where are we with deep brain stimulation? A review of scientific publications and ongoing research. Stereotact Funct Neurosurg. (2022) 100:184–97. doi: 10.1159/000521372, PMID: 35104819

[16] Boogers A Fasano A. A transatlantic viewpoint on the role of pallidal stimulation for Parkinson's disease. Mov Disord. (2024) 39:36–9. doi: 10.1002/mds.29656, PMID: 37965914

[17] Wang X Xiong Y Lin J Lou X. Target selection for magnetic resonance-guided focused ultrasound in the treatment of Parkinson's disease. J Magn Reson Imaging. (2022) 56:35–44. doi: 10.1002/jmri.28080, PMID: 35081263

[18] Xu Y He Q Wang M Gao Y Liu X Li D. Safety and efficacy of magnetic resonance imaging-guided focused ultrasound neurosurgery for Parkinson's disease: a systematic review. Neurosurg Rev. (2021) 44:115–27. doi: 10.1007/s10143-019-01216-y, PMID: 31814058

[19] Tan ZG Zhou Q Huang T Jiang Y. Efficacies of globus pallidus stimulation and subthalamic nucleus stimulation for advanced Parkinson's disease: a meta-analysis of randomized controlled trials. Clin Interv Aging. (2016) 11:777–86. doi: 10.2147/CIA.S105505, PMID: 27382262 PMC4922790

[20] Weaver FM Follett KA Stern M Luo P Harris CL Hur K. Randomized trial of deep brain stimulation for Parkinson disease: thirty-six-month outcomes. Neurology. (2012) 79:55–65. doi: 10.1212/WNL.0b013e31825dcdc1, PMID: 22722632 PMC3385495

[21] Mao Z Ling Z Pan L Xu X Cui Z Liang S. Comparison of efficacy of deep brain stimulation of different targets in Parkinson’s disease: a network meta-analysis. Front Aging Neurosci. (2019) 11:23. doi: 10.3389/fnagi.2019.00023, PMID: 30853908 PMC6395396

[22] Rughani A Schwalb JM Sidiropoulos C Pilitsis J Ramirez-Zamora A Sweet JA. Congress of neurological surgeons systematic review and evidence-based guideline on subthalamic nucleus and globus pallidus internus deep brain stimulation for the treatment of patients with Parkinson's disease: executive summary. Neurosurgery. (2018) 82:753–6. doi: 10.1093/neuros/nyy037, PMID: 29538685 PMC6636249

[23] Odekerken VJ van Laar T Staal MJ Mosch A Hoffmann CFE Nijssen PCG. Subthalamic nucleus versus globus pallidus bilateral deep brain stimulation for advanced Parkinson’s disease (NSTAPS study): a randomised controlled trial. Lancet Neurol. (2013) 12:37–44. doi: 10.1016/S1474-4422(12)70264-8, PMID: 23168021

[24] Reich MM Hsu J Ferguson M Schaper FLWVJ Joutsa J Roothans J. A brain network for deep brain stimulation induced cognitive decline in Parkinson's disease. Brain. (2022) 145:1410–21. doi: 10.1093/brain/awac012, PMID: 35037938 PMC9129093

[25] Obeso JA Marin C Rodriguez-Oroz C Blesa J Benitez-Temiño B Mena-Segovia J. The basal ganglia in Parkinson's disease: current concepts and unexplained observations. Ann Neurol. (2008) 64:S30–46. doi: 10.1002/ana.21481 19127584

[26] Patel DM Walker HC Brooks R Omar N Ditty B Guthrie BL. Adverse events associated with deep brain stimulation for movement disorders: analysis of 510 consecutive cases. Neurosurgery. (2015) 11:190–9. doi: 10.1227/NEU.0000000000000659, PMID: 25599204 PMC12129402

[27] Boutet A Chow CT Narang K Elias GJB Neudorfer C Germann J. Improving Safety of MRI in Patients with Deep Brain Stimulation Devices. Radiology. (2020) 296:250–62. doi: 10.1148/radiol.2020192291, PMID: 32573388 PMC7543718

[28] Olson MC Shill H Ponce F Aslam S. Deep brain stimulation in PD: risk of complications, morbidity, and hospitalizations: a systematic review. Front Aging Neurosci. (2023) 15:1258190. doi: 10.3389/fnagi.2023.1258190, PMID: 38046469 PMC10690827

[29] Moosa S Martínez-Fernández R Elias WJ Del Alamo M Eisenberg HM Fishman PS. The role of high-intensity focused ultrasound as a symptomatic treatment for Parkinson's disease. Mov Disord. (2019) 34:1243–51. doi: 10.1002/mds.27779, PMID: 31291491

[30] Chen KS Chen R. Invasive and noninvasive brain stimulation in Parkinson’s disease: clinical effects and future perspectives. Clin Pharmacol Ther. (2019) 106:763–75. doi: 10.1002/cpt.1542, PMID: 31179534

[31] Jung NY Chang JW. Magnetic resonance-guided focused ultrasound in neurosurgery: taking lessons from the past to inform the future. J Korean Med Sci. (2018) 33:e279. doi: 10.3346/jkms.2018.33.e279, PMID: 30369860 PMC6200905

[32] Schlesinger I Eran A Sinai A Erikh I Nassar M Goldsher D. MRI guided focused ultrasound thalamotomy for moderate-to-severe tremor in Parkinson's disease. Parkinsons Dis. (2015) 2015:219149:1–4. doi: 10.1155/2015/219149, PMID: 26421209 PMC4572440

[33] Polyakova Z Chiken S Hatanaka N Nambu A. Cortical control of subthalamic neuronal activity through the hyperdirect and indirect pathways in monkeys. J Neurosci. (2020) 40:7451–63. doi: 10.1523/JNEUROSCI.0772-20.2020, PMID: 32847963 PMC7511188

[34] Sharma VD Patel M Miocinovic S. Surgical treatment of Parkinson's disease: devices and lesion approaches. Neurotherapeutics. (2020) 17:1525–38. doi: 10.1007/s13311-020-00939-x, PMID: 33118132 PMC7851282

[35] Martínez-Fernández R Rodríguez-Rojas R Del Álamo M Hernández-Fernández F Pineda-Pardo JA Dileone M. Focused ultrasound subthalamotomy in patients with asymmetric Parkinson's disease: a pilot study. Lancet Neurol. (2018) 17:54–63. doi: 10.1016/S1474-4422(17)30403-9, PMID: 29203153

[36] Jung NY Park CK Kim M Lee PH Sohn YH Chang JW. The efficacy and limits of magnetic resonance-guided focused ultrasound pallidotomy for Parkinson's disease: a Phase I clinical trial. J Neurosurg. (2019) 130:1853–1861. doi: 10.3171/2018.2.JNS172514 30095337

[37] Zaaroor M Sinai A Goldsher D Eran A Nassar M Schlesinger I. Magnetic resonance-guided focused ultrasound thalamotomy for tremor: a report of 30 Parkinson’s disease and essential tremor cases. J Neurosurg. (2018) 128:202–10. doi: 10.3171/2016.10.JNS16758, PMID: 28298022

[38] Golfrè Andreasi N Cilia R Romito LM Bonvegna S Straccia G Elia AE. Magnetic resonance-guided focused ultrasound thalamotomy may spare dopaminergic therapy in early-stage tremor-dominant Parkinson's disease: a pilot study. Mov Disord. (2022) 37:2289–95. doi: 10.1002/mds.29200, PMID: 36036203 PMC9804690

[39] Zeng Z Wang L Shi W Xu L Lin Z Xu X. Effects of unilateral stimulation in Parkinson's disease: a randomized double-blind crossover trial. Front Neurol. (2022) 12:12. doi: 10.3389/fneur.2021.812455, PMID: 35126302 PMC8812849

[40] Jost ST Ray Chaudhuri K Ashkan K Loehrer PA Silverdale M Rizos A. Subthalamic stimulation improves quality of sleep in parkinson disease: a 36-month controlled study. J Parkinsons Dis. (2021) 11:323–35. doi: 10.3233/JPD-202278, PMID: 33074192

[41] Dafsari HS Dos Santos Ghilardi MG Visser-Vandewalle V Rizos A Ashkan K Silverdale M. Beneficial nonmotor effects of subthalamic and pallidal neurostimulation in Parkinson's disease. Brain Stimul. (2020) 13:1697–705. doi: 10.1016/j.brs.2020.09.019, PMID: 33038595

[42] Hacker ML Turchan M Heusinkveld LE Currie AD Millan SH Molinari AL. Deep brain stimulation in early-stage Parkinson disease: five-year outcomes. Neurology. (2020) 95:e393–401. doi: 10.1212/WNL.0000000000009946, PMID: 32601120 PMC7455319

[43] Li H Liang S Yu Y Wang Y Cheng Y Yang H. Effect of subthalamic nucleus deep brain stimulation (STN-DBS) on balance performance in Parkinson’s disease. PLoS One. (2020) 15:e0238936. doi: 10.1371/journal.pone.0238936, PMID: 32915893 PMC7486080

[44] Martínez-Fernández R Máñez-Miró JU Rodríguez-Rojas R del Álamo M Shah BB Hernández-Fernández F. Randomized trial of focused ultrasound subthalamotomy for Parkinson's disease. N Engl J Med. (2020) 383:2501–13. doi: 10.1056/NEJMoa2016311, PMID: 33369354

[45] Martinez-Martin P Deuschl G Tonder L Schnitzler A Houeto JL Timmermann L. Interpretation of health-related quality of life outcomes in Parkinson’s disease from the EARLYSTIM Study. PLoS One. (2020) 15:e0237498. doi: 10.1371/journal.pone.0237498, PMID: 32822437 PMC7442251

[46] Vitek JL Jain R Chen L Tröster AI Schrock LE House PA. Subthalamic nucleus deep brain stimulation with a multiple independent constant current-controlled device in Parkinson's disease (INTREPID): a multicentre, double-blind, randomised, sham-controlled study. Lancet Neurol. (2020) 19:491–501. doi: 10.1016/S1474-4422(20)30108-3, PMID: 32470421

[47] Zhang C Wang L Hu W Wang T Zhao Y Pan Y. Combined unilateral subthalamic nucleus and contralateral globus pallidus interna deep brain stimulation for treatment of Parkinson disease: a pilot study of symptom-tailored stimulation. Neurosurgery. (2020) 87:1139–47. doi: 10.1093/neuros/nyaa201, PMID: 32459849 PMC7666906

[48] Valldeoriola F Muñoz E Rumià J Roldán P Cámara A Compta Y. Simultaneous low-frequency deep brain stimulation of the substantia nigra pars reticulata and high-frequency stimulation of the subthalamic nucleus to treat levodopa unresponsive freezing of gait in Parkinson's disease: a pilot study. Parkinsonism Relat Disord. (2019) 60:153–7. doi: 10.1016/j.parkreldis.2018.09.008, PMID: 30241951

[49] Blomstedt P Stenmark Persson R Hariz GM Linder J Fredricks A Häggström B. Deep brain stimulation in the caudal zona incerta versus best medical treatment in patients with Parkinson's disease: a randomised blinded evaluation. J Neurol Neurosurg Psychiatry. (2018) 89:710–6. doi: 10.1136/jnnp-2017-317219, PMID: 29386253 PMC6031280

[50] Gratwicke J Zrinzo L Kahan J Peters A Beigi M Akram H. Bilateral deep brain stimulation of the nucleus basalis of meynert for Parkinson disease dementia: a randomized clinical trial. JAMA Neurol. (2018) 75:169–78. doi: 10.1001/jamaneurol.2017.3762, PMID: 29255885 PMC5838617

[51] Bond AE Shah BB Huss DS Dallapiazza RF Warren A Harrison MB. Safety and efficacy of focused ultrasound thalamotomy for patients with medication-refractory, tremor-dominant Parkinson disease: a randomized clinical trial. JAMA Neurol. (2017) 74:1412–8. doi: 10.1001/jamaneurol.2017.3098, PMID: 29084313 PMC5822192

[52] Hacker ML Tonascia J Turchan M Currie A Heusinkveld L Konrad PE. Deep brain stimulation may reduce the relative risk of clinically important worsening in early stage Parkinson's disease. Parkinsonism Relat Disord. (2015) 21:1177–83. doi: 10.1016/j.parkreldis.2015.08.008, PMID: 26306000

[53] St George RJ Carlson-Kuhta P King LA Burchiel KJ Horak FB. Compensatory stepping in Parkinson's disease is still a problem after deep brain stimulation randomized to STN or GPi. J Neurophysiol. (2015) 114:1417–23. doi: 10.1152/jn.01052.2014, PMID: 26108960 PMC4556849

[54] Charles D Konrad PE Neimat JS Molinari AL Tramontana MG Finder SG. Subthalamic nucleus deep brain stimulation in early stage Parkinson's disease. Parkinsonism Relat Disord. (2014) 20:731–7. doi: 10.1016/j.parkreldis.2014.03.019, PMID: 24768120 PMC4103427

[55] Okun MS Wu SS Fayad S Ward H Bowers D Rosado C. Acute and chronic mood and apathy outcomes from a randomized study of unilateral STN and GPi DBS. PLoS One. (2014) 9:e114140. doi: 10.1371/journal.pone.0114140, PMID: 25469706 PMC4254912

[56] Chang C Li N Wu Y Geng N Ge S Wang J. Associations between bilateral subthalamic nucleus deep brain stimulation (STN-DBS) and Anxiety in Parkinson’s disease patients: a controlled study. J Neuropsychiatry Clin Neurosci. (2012) 24:316–25. doi: 10.1176/appi.neuropsych.11070170, PMID: 23037645

[57] Okun MS Gallo BV Mandybur G Jagid J Foote KD Revilla FJ. Subthalamic deep brain stimulation with a constant-current device in Parkinson’s disease: an open-label randomised controlled trial. Lancet Neurol. (2012) 11:140–9. doi: 10.1016/S1474-4422(11)70308-8, PMID: 22239915

[58] Rocchi L Carlson-Kuhta P Chiari L Burchiel KJ Hogarth P Horak FB. Effects of deep brain stimulation in the subthalamic nucleus or globus pallidus internus on step initiation in Parkinson disease: laboratory investigation. J Neurosurg. (2012) 117:1141–9. doi: 10.3171/2012.8.JNS112006, PMID: 23039143 PMC3990225

[59] Robertson LT St George RJ Carlson-Kuhta P Hogarth P Burchiel KJ Horak FB. Site of deep brain stimulation and jaw velocity in Parkinson disease: clinical article. J Neurosurg. (2011) 115:985–94. doi: 10.3171/2011.7.JNS102173, PMID: 21838506 PMC3517909

[60] Smeding HMM Speelman JD Huizenga HM Schuurman PR Schmand B. Predictors of cognitive and psychosocial outcome after STN DBS in Parkinson’s disease. J Neurol Neurosurg Psychiatry. (2011) 82:754–60. doi: 10.1136/jnnp.2007.140012, PMID: 19465417

[61] Follett KA Weaver FM Stern M Hur K Harris CL Luo P. Pallidal versus subthalamic deep-brain stimulation for Parkinson's disease. N Engl J Med. (2010) 362:2077–91. doi: 10.1056/NEJMoa0907083, PMID: 20519680

[62] Moro E Lozano AM Pollak P Agid Y Rehncrona S Volkmann J. Long-term results of a multicenter study on subthalamic and pallidal stimulation in Parkinson's disease. Mov Disord. (2010) 25:578–86. doi: 10.1002/mds.22735, PMID: 20213817

[63] Montel SR Bungener C. Coping and quality of life of patients with Parkinson disease who have undergone deep brain stimulation of the subthalamic nucleus. Surg Neurol. (2009) 72:105–10. doi: 10.1016/j.surneu.2008.05.026, PMID: 18786708

[64] Volkmann J Albanese A Kulisevsky J Tornqvist AL Houeto JL Pidoux B. Long-term effects of pallidal or subthalamic deep brain stimulation on quality of life in Parkinson's disease. Mov Disord. (2009) 24:1154–61. doi: 10.1002/mds.22496, PMID: 19412954

[65] Zahodne LB Okun MS Foote KD Fernandez HH Rodriguez RL Wu SS. Greater improvement in quality of life following unilateral deep brain stimulation surgery in the globus pallidus as compared to the subthalamic nucleus. J Neurol. (2009) 256:1321–9. doi: 10.1007/s00415-009-5121-7, PMID: 19363633 PMC3045861

[66] Zangaglia R Pacchetti C Pasotti C Mancini F Servello D Sinforiani E. Deep brain stimulation and cognitive functions in Parkinson's disease: a three-year controlled study. Mov Disord. (2009) 24:1621–8. doi: 10.1002/mds.22603, PMID: 19514093

[67] Witt K Daniels C Reiff J Krack P Volkmann J Pinsker MO. Neuropsychological and psychiatric changes after deep brain stimulation for Parkinson's disease: a randomised, multicentre study. Lancet Neurol. (2008) 7:605–14. doi: 10.1016/S1474-4422(08)70114-5, PMID: 18538636

[68] Deuschl G Schade-Brittinger C Krack P Volkmann J Schäfer H Bötzel K. A randomized trial of deep-brain stimulation for Parkinson's disease. N Engl J Med. (2006) 355:896–908. doi: 10.1056/NEJMoa060281, PMID: 16943402

[69] Anderson VC Burchiel KJ Hogarth P Favre J Hammerstad JP. Pallidal vs subthalamic nucleus deep brain stimulation in Parkinson disease. Arch Neurol. (2005) 62:554–60. doi: 10.1001/archneur.62.4.554 15824252

[70] Capecci M Ricciuti RA Burini D Bombace VG Provinciali L Iacoangeli M. Functional improvement after subthalamic stimulation in Parkinson's disease: a non-equivalent controlled study with 12-24 month follow up. J Neurol Neurosurg Psychiatry. (2005) 76:769–74. doi: 10.1136/jnnp.2004.047001, PMID: 15897496 PMC1739649

[71] Just H Ostergaard K. Health-related quality of life in patients with advanced Parkinson's disease treated with deep brain stimulation of the subthalamic nuclei. Mov Disord. (2002) 17:539–45. doi: 10.1002/mds.10111, PMID: 12112204

