# Supplementary material for: Ameliorating motor performance and quality of life in Parkinson’s disease: a comparison of deep brain stimulation and focused ultrasound surgery
Source: Front Neurol. 2025 Apr 30;16:1449973. doi: 10.3389/fneur.2025.1449973 (PMC12074956; doi:10.3389/fneur.2025.1449973)
Supplement: Supplementary file 1 [file Data_Sheet_1.docx]

Supplementary sheet 1. Search strategy for PubMed

| Search number | Query | Sort By | Filters | Search Details | Results | Time |
| --- | --- | --- | --- | --- | --- | --- |
| 10 | ((((((((((deep brain stimulation[MeSH Terms]) OR (Deep Brain Stimulations[Title/Abstract])) OR (Deep Brain Stimulations[Title/Abstract])) OR (Stimulation, Deep Brain[Title/Abstract])) OR (Stimulations, Deep Brain[Title/Abstract])) OR (Brain Stimulation, Deep[Title/Abstract])) OR (Electrical Stimulation of the Brain[Title/Abstract])) OR (dbs[Title/Abstract])) AND ((((((quality of life[Title/Abstract]) OR (QOL[Title/Abstract])) OR (Life Quality[Title/Abstract])) OR (Health-Related Quality Of Life[Title/Abstract])) OR (Health Related Quality Of Life[Title/Abstract])) OR (HRQOL[Title/Abstract]))) OR (((((((((((focused ultrasound[Title/Abstract]) OR (focused ultrasound stimulation[Title/Abstract])) OR (MRIgFUS[Title/Abstract])) OR (MRI-guided focused ultrasound[Title/Abstract])) OR (MRgFUS[Title/Abstract])) OR (magnetic resonance–guided focused ultrasound[Title/Abstract])) OR (MR-guided focused ultrasound[Title/Abstract])) OR (magnetic resonance guided high-intensity focused ultrasound[Title/Abstract])) OR (MRI-guided FUS ablation[Title/Abstract])) OR (fus[Title/Abstract])))) AND (((((((((((((parkinson disease[MeSH Terms]) OR (Parkinson's Disease[Title/Abstract])) OR (Idiopathic Parkinson's Disease[Title/Abstract])) OR (Idiopathic Parkinson Disease[Title/Abstract])) OR (Parkinson's Disease, Idiopathic[Title/Abstract])) OR (Parkinson Disease, Idiopathic[Title/Abstract])) OR (Lewy Body Parkinson's Disease[Title/Abstract])) OR (Lewy Body Parkinson Disease[Title/Abstract])) OR (Parkinson's Disease, Lewy Body[Title/Abstract])) OR (Primary Parkinsonism[Title/Abstract])) OR (Parkinsonism, Primary[Title/Abstract])) OR (Paralysis Agitans[Title/Abstract])) OR (parkinsons disease[Title/Abstract])) |  |  | ((("deep brain stimulation"[MeSH Terms] OR "deep brain stimulations"[Title/Abstract] OR "deep brain stimulations"[Title/Abstract] OR "stimulation deep brain"[Title/Abstract] OR (("stimulate"[All Fields] OR "stimulated"[All Fields] OR "stimulates"[All Fields] OR "stimulating"[All Fields] OR "Stimulation"[All Fields] OR "Stimulations"[All Fields] OR "stimulative"[All Fields] OR "stimulator"[All Fields] OR "stimulator s"[All Fields] OR "stimulators"[All Fields]) AND "deep brain"[Title/Abstract]) OR "brain stimulation deep"[Title/Abstract] OR "electrical stimulation of the brain"[Title/Abstract] OR "dbs"[Title/Abstract]) AND ("quality of life"[Title/Abstract] OR "QOL"[Title/Abstract] OR "life quality"[Title/Abstract] OR "health related quality of life"[Title/Abstract] OR "health related quality of life"[Title/Abstract] OR "HRQOL"[Title/Abstract])) OR ("focused ultrasound"[Title/Abstract] OR "focused ultrasound stimulation"[Title/Abstract] OR "MRIgFUS"[Title/Abstract] OR "mri guided focused ultrasound"[Title/Abstract] OR "MRgFUS"[Title/Abstract] OR "magnetic resonance guided focused ultrasound"[Title/Abstract] OR "mr guided focused ultrasound"[Title/Abstract] OR "magnetic resonance guided high intensity focused ultrasound"[Title/Abstract] OR ("MRI-guided"[All Fields] AND "fus ablation"[Title/Abstract]) OR "FUS"[Title/Abstract])) AND ("parkinson disease"[MeSH Terms] OR "parkinson s disease"[Title/Abstract] OR "idiopathic parkinson s disease"[Title/Abstract] OR "idiopathic parkinson disease"[Title/Abstract] OR "parkinson s disease idiopathic"[Title/Abstract] OR "parkinson disease idiopathic"[Title/Abstract] OR "lewy body parkinson s disease"[Title/Abstract] OR "lewy body parkinson disease"[Title/Abstract] OR "parkinson s disease lewy body"[Title/Abstract] OR "primary parkinsonism"[Title/Abstract] OR "parkinsonism primary"[Title/Abstract] OR "paralysis agitans"[Title/Abstract] OR "parkinsons disease"[Title/Abstract]) | 960 | 8:03:56 |
| 9 | (((((((((deep brain stimulation[MeSH Terms]) OR (Deep Brain Stimulations[Title/Abstract])) OR (Deep Brain Stimulations[Title/Abstract])) OR (Stimulation, Deep Brain[Title/Abstract])) OR (Stimulations, Deep Brain[Title/Abstract])) OR (Brain Stimulation, Deep[Title/Abstract])) OR (Electrical Stimulation of the Brain[Title/Abstract])) OR (dbs[Title/Abstract])) AND ((((((quality of life[Title/Abstract]) OR (QOL[Title/Abstract])) OR (Life Quality[Title/Abstract])) OR (Health-Related Quality Of Life[Title/Abstract])) OR (Health Related Quality Of Life[Title/Abstract])) OR (HRQOL[Title/Abstract]))) OR (((((((((((focused ultrasound[Title/Abstract]) OR (focused ultrasound stimulation[Title/Abstract])) OR (MRIgFUS[Title/Abstract])) OR (MRI-guided focused ultrasound[Title/Abstract])) OR (MRgFUS[Title/Abstract])) OR (magnetic resonance–guided focused ultrasound[Title/Abstract])) OR (MR-guided focused ultrasound[Title/Abstract])) OR (magnetic resonance guided high-intensity focused ultrasound[Title/Abstract])) OR (MRI-guided FUS ablation[Title/Abstract])) OR (fus[Title/Abstract]))) |  |  | (("deep brain stimulation"[MeSH Terms] OR "deep brain stimulations"[Title/Abstract] OR "deep brain stimulations"[Title/Abstract] OR "stimulation deep brain"[Title/Abstract] OR (("stimulate"[All Fields] OR "stimulated"[All Fields] OR "stimulates"[All Fields] OR "stimulating"[All Fields] OR "Stimulation"[All Fields] OR "Stimulations"[All Fields] OR "stimulative"[All Fields] OR "stimulator"[All Fields] OR "stimulator s"[All Fields] OR "stimulators"[All Fields]) AND "deep brain"[Title/Abstract]) OR "brain stimulation deep"[Title/Abstract] OR "electrical stimulation of the brain"[Title/Abstract] OR "dbs"[Title/Abstract]) AND ("quality of life"[Title/Abstract] OR "QOL"[Title/Abstract] OR "life quality"[Title/Abstract] OR "health related quality of life"[Title/Abstract] OR "health related quality of life"[Title/Abstract] OR "HRQOL"[Title/Abstract])) OR ("focused ultrasound"[Title/Abstract] OR "focused ultrasound stimulation"[Title/Abstract] OR "MRIgFUS"[Title/Abstract] OR "mri guided focused ultrasound"[Title/Abstract] OR "MRgFUS"[Title/Abstract] OR "magnetic resonance guided focused ultrasound"[Title/Abstract] OR "mr guided focused ultrasound"[Title/Abstract] OR "magnetic resonance guided high intensity focused ultrasound"[Title/Abstract] OR ("MRI-guided"[All Fields] AND "fus ablation"[Title/Abstract]) OR "FUS"[Title/Abstract]) | 11,584 | 8:03:40 |
| 8 | ((((((((deep brain stimulation[MeSH Terms]) OR (Deep Brain Stimulations[Title/Abstract])) OR (Deep Brain Stimulations[Title/Abstract])) OR (Stimulation, Deep Brain[Title/Abstract])) OR (Stimulations, Deep Brain[Title/Abstract])) OR (Brain Stimulation, Deep[Title/Abstract])) OR (Electrical Stimulation of the Brain[Title/Abstract])) OR (dbs[Title/Abstract])) AND ((((((quality of life[Title/Abstract]) OR (QOL[Title/Abstract])) OR (Life Quality[Title/Abstract])) OR (Health-Related Quality Of Life[Title/Abstract])) OR (Health Related Quality Of Life[Title/Abstract])) OR (HRQOL[Title/Abstract])) |  |  | ("deep brain stimulation"[MeSH Terms] OR "deep brain stimulations"[Title/Abstract] OR "deep brain stimulations"[Title/Abstract] OR "stimulation deep brain"[Title/Abstract] OR (("stimulate"[All Fields] OR "stimulated"[All Fields] OR "stimulates"[All Fields] OR "stimulating"[All Fields] OR "Stimulation"[All Fields] OR "Stimulations"[All Fields] OR "stimulative"[All Fields] OR "stimulator"[All Fields] OR "stimulator s"[All Fields] OR "stimulators"[All Fields]) AND "deep brain"[Title/Abstract]) OR "brain stimulation deep"[Title/Abstract] OR "electrical stimulation of the brain"[Title/Abstract] OR "dbs"[Title/Abstract]) AND ("quality of life"[Title/Abstract] OR "QOL"[Title/Abstract] OR "life quality"[Title/Abstract] OR "health related quality of life"[Title/Abstract] OR "health related quality of life"[Title/Abstract] OR "HRQOL"[Title/Abstract]) | 1,243 | 8:03:13 |
| 7 | ((((((((((((parkinson disease[MeSH Terms]) OR (Parkinson's Disease[Title/Abstract])) OR (Idiopathic Parkinson's Disease[Title/Abstract])) OR (Idiopathic Parkinson Disease[Title/Abstract])) OR (Parkinson's Disease, Idiopathic[Title/Abstract])) OR (Parkinson Disease, Idiopathic[Title/Abstract])) OR (Lewy Body Parkinson's Disease[Title/Abstract])) OR (Lewy Body Parkinson Disease[Title/Abstract])) OR (Parkinson's Disease, Lewy Body[Title/Abstract])) OR (Primary Parkinsonism[Title/Abstract])) OR (Parkinsonism, Primary[Title/Abstract])) OR (Paralysis Agitans[Title/Abstract])) OR (parkinsons disease[Title/Abstract]) |  |  | "parkinson disease"[MeSH Terms] OR "parkinson s disease"[Title/Abstract] OR "idiopathic parkinson s disease"[Title/Abstract] OR "idiopathic parkinson disease"[Title/Abstract] OR "parkinson s disease idiopathic"[Title/Abstract] OR "parkinson disease idiopathic"[Title/Abstract] OR "lewy body parkinson s disease"[Title/Abstract] OR "lewy body parkinson disease"[Title/Abstract] OR "parkinson s disease lewy body"[Title/Abstract] OR "primary parkinsonism"[Title/Abstract] OR "parkinsonism primary"[Title/Abstract] OR "paralysis agitans"[Title/Abstract] OR "parkinsons disease"[Title/Abstract] | 123,671 | 8:00:23 |
| 6 | ((((((((((focused ultrasound[Title/Abstract]) OR (focused ultrasound stimulation[Title/Abstract])) OR (MRIgFUS[Title/Abstract])) OR (MRI-guided focused ultrasound[Title/Abstract])) OR (MRgFUS[Title/Abstract])) OR (magnetic resonance–guided focused ultrasound[Title/Abstract])) OR (MR-guided focused ultrasound[Title/Abstract])) OR (magnetic resonance guided high-intensity focused ultrasound[Title/Abstract])) OR (MRI-guided FUS ablation[Title/Abstract])) OR (fus[Title/Abstract])) |  |  | "focused ultrasound"[Title/Abstract] OR "focused ultrasound stimulation"[Title/Abstract] OR "MRIgFUS"[Title/Abstract] OR "mri guided focused ultrasound"[Title/Abstract] OR "MRgFUS"[Title/Abstract] OR "magnetic resonance guided focused ultrasound"[Title/Abstract] OR "mr guided focused ultrasound"[Title/Abstract] OR "magnetic resonance guided high intensity focused ultrasound"[Title/Abstract] OR ("MRI-guided"[All Fields] AND "fus ablation"[Title/Abstract]) OR "FUS"[Title/Abstract] | 10,369 | 7:55:02 |
| 4 | (((((quality of life[Title/Abstract]) OR (QOL[Title/Abstract])) OR (Life Quality[Title/Abstract])) OR (Health-Related Quality Of Life[Title/Abstract])) OR (Health Related Quality Of Life[Title/Abstract])) OR (HRQOL[Title/Abstract]) |  |  | "quality of life"[Title/Abstract] OR "QOL"[Title/Abstract] OR "life quality"[Title/Abstract] OR "health related quality of life"[Title/Abstract] OR "health related quality of life"[Title/Abstract] OR "HRQOL"[Title/Abstract] | 358,346 | 7:53:44 |
| 3 | (((((((deep brain stimulation[MeSH Terms]) OR (Deep Brain Stimulations[Title/Abstract])) OR (Deep Brain Stimulations[Title/Abstract])) OR (Stimulation, Deep Brain[Title/Abstract])) OR (Stimulations, Deep Brain[Title/Abstract])) OR (Brain Stimulation, Deep[Title/Abstract])) OR (Electrical Stimulation of the Brain[Title/Abstract])) OR (dbs[Title/Abstract]) |  |  | "deep brain stimulation"[MeSH Terms] OR "deep brain stimulations"[Title/Abstract] OR "deep brain stimulations"[Title/Abstract] OR "stimulation deep brain"[Title/Abstract] OR (("stimulate"[All Fields] OR "stimulated"[All Fields] OR "stimulates"[All Fields] OR "stimulating"[All Fields] OR "Stimulation"[All Fields] OR "Stimulations"[All Fields] OR "stimulative"[All Fields] OR "stimulator"[All Fields] OR "stimulator s"[All Fields] OR "stimulators"[All Fields]) AND "deep brain"[Title/Abstract]) OR "brain stimulation deep"[Title/Abstract] OR "electrical stimulation of the brain"[Title/Abstract] OR "dbs"[Title/Abstract] | 21,203 | 7:51:17 |

Supplementary sheet 2. Search strategy for Embase

Embase

Session Results

.......................................................

No. Query Results Results Date

#15. #3 AND #14 2,590 20 Jan 2023

#14. #10 OR #13 21,090 20 Jan 2023

#13. #11 OR #12 17,501 20 Jan 2023

#12. 'focused ultrasonic ablation':ti,ab,kw OR 15,232 20 Jan 2023

'focused ultrasonic irradiation':ti,ab,kw OR

'focused ultrasound':ti,ab,kw OR 'focused

ultrasound treatment':ti,ab,kw OR fus:ti,ab,kw

#11. 'focused ultrasound therapy'/exp 8,470 20 Jan 2023

#10. #6 AND #9 3,651 20 Jan 2023

#9. #7 OR #8 641,199 20 Jan 2023

#8. 'health related quality of life':ti,ab,kw OR 181,448 20 Jan 2023

hrql:ti,ab,kw OR 'life quality':ti,ab,kw OR

qol:ti,ab,kw

#7. 'quality of life'/exp 616,512 20 Jan 2023

#6. #4 OR #5 64,367 20 Jan 2023

#5. 'brain excitation':ti,ab,kw OR 'brain 40,838 20 Jan 2023

stimulation':ti,ab,kw OR 'brain

stimulus':ti,ab,kw OR 'deep brain

stimulation':ti,ab,kw OR 'electrical brain

stimulation':ti,ab,kw OR 'excitation,

brain':ti,ab,kw OR dbs:ti,ab,kw

#4. 'brain depth stimulation'/exp 50,454 20 Jan 2023

#3. #1 OR #2 212,552 20 Jan 2023

#2. 'idiopathic parkinsonism':ti,ab,kw OR 'lewy 154,973 20 Jan 2023

bodies of parkinson disease':ti,ab,kw OR 'lewy

bodies of parkinson`s disease':ti,ab,kw OR 'lewy

bodies of parkinsons disease':ti,ab,kw OR 'lewy

body parkinson disease':ti,ab,kw OR 'lewy body

parkinson`s disease':ti,ab,kw OR 'lewy body

parkinsons disease':ti,ab,kw OR 'paralysis

agitans':ti,ab,kw OR 'parkinson dementia

complex':ti,ab,kw OR 'parkinson`s

disease':ti,ab,kw OR 'parkinsons

disease':ti,ab,kw OR 'primary

parkinsonism':ti,ab,kw

#1. 'parkinson disease'/exp 188,181 20 Jan 2023

.......................................................

Supplementary sheet 3. Search strategy for Cochrane

Search Name:

Date Run: 20/01/2023 14:16:50

Comment:

ID Search Hits

#1 MeSH descriptor: [Parkinson Disease] explode all trees 4844

#2 (Parkinson's Disease):ti,ab,kw OR (Idiopathic Parkinson's Disease):ti,ab,kw OR (Idiopathic Parkinson Disease):ti,ab,kw AND (Parkinson's Disease, Idiopathic):ti,ab,kw AND (Parkinson Disease, Idiopathic):ti,ab,kw 11687

#3 (Lewy Body Parkinson's Disease):ti,ab,kw OR (Lewy Body Parkinson Disease):ti,ab,kw OR (Parkinson's Disease, Lewy Body):ti,ab,kw OR (Primary Parkinsonism):ti,ab,kw OR (Parkinsonism, Primary):ti,ab,kw 544

#4 (Paralysis Agitans):ti,ab,kw OR (parkinsons disease):ti,ab,kw 944

#5 #1 or #2 or #3 or #4 11877

#6 MeSH descriptor: [Deep Brain Stimulation] explode all trees 355

#7 (Brain Stimulations, Deep):ti,ab,kw OR (Deep Brain Stimulations):ti,ab,kw OR (Stimulation, Deep Brain):ti,ab,kw OR (Stimulations, Deep Brain):ti,ab,kw OR (Brain Stimulation, Deep):ti,ab,kw 1665

#8 (Electrical Stimulation of the Brain):ti,ab,kw OR (DBS):ti,ab,kw 2760

#9 #6 or #7 or #8 3315

#10 (focused ultrasound):ti,ab,kw OR (focused ultrasound stimulation):ti,ab,kw OR (MRIgFUS):ti,ab,kw OR (MRI-guided focused ultrasound):ti,ab,kw OR (MRgFUS):ti,ab,kw 913

#11 (magnetic resonance–guided focused ultrasound):ti,ab,kw OR (MR-guided focused ultrasound):ti,ab,kw OR (magnetic resonance guided high-intensity focused ultrasound):ti,ab,kw OR (MRI-guided FUS ablation):ti,ab,kw OR (fus):ti,ab,kw 235

#12 #10 or #11 1005

#13 #9 or #12 4294

#14 #5 and #13 954

Supplementary sheet 4. Adverse event

| Number | Author&Year | Treatment | Adverse events |
| --- | --- | --- | --- |
| 1 | Krishna2023 | GPi_FUS | Pallidotomy-related(Decreased biceps reflex, Decreased foot vibration, Dysarthria, Gait disturbance, Loss of taste, Visual disturbance, Facial weakness, Other); Parkinson’s disease–related(Difficulty walking or imbalance, Fall, Leg cramp or dystonia, Musculoskeletal, Nonmotor symptoms, Numbness or tingling, Reduced verbal fluency); Procedure-related(Fatigue, Frame or pin-site complications, Headache, Pulmonary embolism, Sonication-related pain, Vertigo or dizziness) |
|  |  | MT* | Parkinson’s disease–related(Dizziness); Procedure-related |
| 2 | Andreasi2022 | VIM_FUS | Dopaminergic therapy-related(Motor Fluctuations, Impulse control disorder, Leg edema); Thalamotomy Related(Orofacial paresthesias, Ataxia, Hemiparesis, Dysarthria); MRI or Ultrasonography Related(Nausea/vomit, Scalp Numbness, Dizziness, Headache, Uncomfortable heat sensation, Anxiety, Neck pain); Unrelated(Low back pain, Sleep apnea disorder) |
|  |  | MT | Dopaminergic therapy-related(Motor Fluctuations, Dyskinesias, Impulse control disorder, Nausea, Leg edema, Hypotension, Daytime sleepiness, Anxiety/agitation, Skin reaction to rotigotine); Unrelated(Low back pain, Prostatic neoplasm, Subjective memory loss) |
| 3 | Weiss2022 | STN_DBS | NA |
|  |  | MT | NA |
| 4 | Zeng2022 | STN_DBS | No adverse events were observed |
|  |  | GPi_DBS | No adverse events were observed |
| 5 | Jost2021 | STN_DBS | NA |
|  |  | MT | NA |
| 6 | Dafsari2020 | STN_DBS | NA |
|  |  | GPi_DBS | NA |
| 7 | Hacker2020 | STN_DBS | NA |
|  |  | MT | NA |
| 8 | Li2020 | STN_DBS | NA |
|  |  | MT | NA |
| 9 | Martínez-Fernández2020 | STN_FUS | Dyskinesia, Weakness, Isolated facial asymmetry, Speech disturbance, Gait disturbance, Somnolence |
|  |  | MT* | No adverse events were observed |
| 10 | Martinez-Martin2020 | STN_DBS | NA |
|  |  | MT | NA |
| 11 | Vitek2020 | STN_DBS | Related to hardware(Post-operative confusion, Infection, Pneumocephalus); Related to procedure(Aphasia, Post-operative confusion, Infection, Hypoventilation, Myocardial infarction, Pneumocephalus, Fever of unknown origin, Wound haemorrhage) |
|  |  | MT* | Related to hardware(Seizure, Infection, Implant site oedema, Intracranial hypotension); Related to procedure(Seizure, Infection, Implant site oedema, Intracranial hypotension) |
| 12 | Zhang2020 | STN-GPi_DBS | No adverse events were observed |
|  |  | MT | No adverse events were observed |
| 13 | Valldeoriola2019 | STN_DBS | Confused state, Blurred vision, Muscular twitching, Depressive symptoms |
|  |  | SNr_DBS | Confused state, Blurred vision, Muscular twitching, Depressive symptoms |
|  |  | STN-SNr_DBS | Confused state, Blurred vision, Muscular twitching, Depressive symptoms |
| 14 | Blomstedt2018 | cZi_DBS | Deep venous thrombosis |
|  |  | MT | No adverse events were observed |
| 15 | Gratwicke2018 | NBM_DBS | Serious adverse events related to surgery or device(Erosion of right electrode cap through scalp); Non‐serious adverse events related to surgery or device(Superficial scalp wound infection, Urethral tear from traumatic catheterisation, Post‐operative transient confusion/paranoia, Burr hole cap discomfort); Non‐serious adverse events related to stimulation(Visual hallucinations); Other(Increased limb rigidity, Worsened postural hypotension, Worsened urinary frequency, Jejunal tube blockage) |
|  |  | MT* | Serious adverse events related to surgery or device(Erosion of right electrode cap through scalp); Non‐serious adverse events related to surgery or device(Superficial scalp wound infection, Urethral tear from traumatic catheterisation, Post‐operative transient confusion/paranoia, Burr hole cap discomfort); Non‐serious adverse events related to stimulation(Visual hallucinations); Other(Increased limb rigidity, Worsened postural hypotension, Worsened urinary frequency, Jejunal tube blockage) |
| 16 | Bond2017 | VIM_FUS | Thalamotomy Related(Finger paresthesia, Orofacial paresthesia, Ataxia, Hemiparesis, Dysmetria, Mild vocal change); MRI or Ultrasonography Related(Scalp numbness, Headache, Dizziness or vertigo, Head pain or heat sensation, Stomach pain or nausea or emesis, Periorbital swelling, Neck or back or shoulder pain, Decline in mental status, Pin site pain, Anxiety, Light headedness, Right-sided ecchymosis, Spot in visual field); Unrelated(Decline in visuospatial abilities, Brief loss of reality, Increased daytime sleepiness, Decreased hand dexterity, Worsening degenerative knee disease, Cholecystitis or cholecystectomy, Worsening of depression) |
|  |  | MT* | MRI or Ultrasonography Related(Headache, Dizziness or vertigo, Stomach pain or nausea or emesis, Neck or back or shoulder pain, Pin site pain) |
| 17 | Hacker2015 | STN_DBS | NA |
|  |  | MT | NA |
| 18 | St George2015 | STN_DBS | NA |
|  |  | GPi_DBS | NA |
|  |  | MT | NA |
| 19 | Charles2014 | STN_DBS | Insomnia, Neck pain, Pain in extremity, Weight decreased, Presyncope, Musculoskeletal pain, Back pain, Bronchitis, Hallucination, Nausea, Pain, Rib fracture, Nasopharyngitis, Depression, Implant site pain |
|  |  | MT | Insomnia, Chest pain, Urinary tract infection, Neck pain, Pain in extremity, Weight decreased, Presyncope, Musculoskeletal pain, Dyspnoea, Vomiting, Dizziness, Back pain, Bronchitis, Hallucination, Nausea, Pain, Rib fracture |
| 20 | Okun2014 | STN_DBS | NA |
|  |  | GPi_DBS | NA |
| 21 | Schuepbach2013 | STN_DBS | Serious adverse events(Death, all by suicide, Life-threatening event); Event related to medication or stimulation(Worsening of mobility, Dyskinesia, Impulse control disorder, Depression, Suicidal ideation, Suicide attempt, Injury, Respiratory or thoracic disorder, Other); Event related to surgery or device(Impaired wound healing, Intracerebral abscess or edema, Dislocation of device, Reoperation necessary, Other); Event related to Parkinson’s disease(Dyskinesia, Gait disorder, Worsening of mobility, Depression, Sleep disorder, Impulse control disorder, Musculoskeletal or connective tissue disorder, Pain, Weight change) |
|  |  | MT | Serious adverse events(Death, all by suicide, Life-threatening event); Event related to medication or stimulation(Worsening of mobility, Motor fluctuations, Dyskinesia, Psychosis or hallucinations, Anxiety, Impulse control disorder, Depression, Suicide attempt, Cardiac disorder, Other); Event related to Parkinson’s disease(Dyskinesia, Gait disorder, Worsening of mobility, Depression, Sleep disorder, Impulse control disorder, Musculoskeletal or connective tissue disorder, Pain, Weight change) |
| 22 | Chang2012 | STN_DBS | NA |
|  |  | MT | NA |
| 23 | Okun2012 | STN_DBS | Serious adverse events(Confusion, CSF leakage, Gait disorder including balance problems, Hardware problem, Infection, Intracranial haemorrhage, Lead migration, Motor fluctuations, Worsening of PD symptoms, Seizures or convulsions, Tremor, Unrelated events); Non-serious adverse events(Anxiety, Confusion, Depression, Disequilibrium, Dysarthria, Dyskinesia, Dysphasia, Dystonia, Oedema, Falls, Fatigue, Gait disorder including balance problems, Hallucinations, Headache, Infection, Jolting or shocking sensation, Paraesthesia, Postoperation pain/stress/discomfort, Psychiatric changes or disturbances, Sleep disturbances, Subcutaneous haemorrhage or seroma, Unrelated events) |
|  |  | MT | Serious adverse events(Infection, Intracranial haemorrhage, Worsening of PD symptoms, Pneumonia, Unrelated events); Non-serious adverse events(Anxiety, Confusion, Disequilibrium, Dyskinesia, Dysphasia, Gait disorder including balance problems, Hallucinations, Headache, Subcutaneous haemorrhage or seroma, Unrelated events) |
| 24 | Rocchi2012 | STN_DBS | NA |
|  |  | GPi_DBS | NA |
| 25 | Weaver2012 | STN_DBS | NA |
|  |  | GPi_DBS | NA |
| 26 | Robertson2011 | STN_DBS | NA |
|  |  | GPi_DBS | NA |
| 27 | Smeding2011 | STN_DBS | Psychiatric events |
|  |  | MT | Depression |
| 28 | Follett2010 | STN_DBS | Serious adverse event(Implantation-site infection, Fall, Pneumonia, Confusional state, Medical-device complication, Lumbar spine stenosis, Mental status change, Osteoarthritis, Syncope, Depression, Adverse drug reaction, Coronary artery disease, Dyskinesia, Gastroesophageal reflux disease, Inguinal hernia, Suicidal depression, Cerebral hemorrhage, Stroke ); Moderate or severe adverse event(Fall, Gait disturbance, Depression, Balance disorder, Speech problem, Freezing phenomenon, Bradykinesia, Motor dysfunction, Dyskinesia, Dystonia, Confused state) |
|  |  | GPi_DBS | Serious adverse event(Implantation-site infection, Fall, Pneumonia, Confusional state, Medical-device complication, Lumbar spine stenosis, Mental status change, Osteoarthritis, Syncope, Depression, Adverse drug reaction, Coronary artery disease, Dyskinesia, Gastroesophageal reflux disease, Inguinal hernia, Suicidal depression, Cerebral hemorrhage, Intracranial hemorrhage); Moderate or severe adverse event(Fall, Gait disturbance, Depression, Balance disorder, Speech problem, Freezing phenomenon, Bradykinesia, Motor dysfunction, Dyskinesia, Dystonia, Confused state) |
| 29 | Moro2010 | STN_DBS | Cognitive decline, Depression/anxiety, Hypersexuality, Speech difficulties, Balance disturbances, Gait disorders, Motor fluctuations, Sleep disorders |
|  |  | GPi_DBS | Cognitive decline, Depression/anxiety, Hypersexuality, Speech difficulties, Balance disturbances, Gait disorders |
| 30 | Montel2009 | STN_DBS | NA |
|  |  | MT | NA |
| 31 | Volkmann2009 | STN_DBS | Neuropsychiatric AE (Memory decline, Psychiatric disturbance, Depression, Apathy, Mood disturbances, Hypersexuality), Speech and swallowing AE (Speech difficulties, Dysphonia, Dysarthria, Dysphagia), Gait and balance AE (Disequilibrium, Falls, Balance disturbance) |
|  |  | GPi_DBS | NA |
| 32 | Zahodne2009 | STN_DBS | NA |
|  |  | GPi_DBS | NA |
| 33 | Zangaglia2009 | STN_DBS | NA |
|  |  | MT | NA |
| 34 | Witt2008 | STN_DBS | NA |
|  |  | MT | NA |
| 35 | Deuschl2006 | STN_DBS | Serious adverse event including Death(Perioperative cerebral hematoma, Suicide 5 mo after surgery, Pneumonia) and Readmission to the hospital(Worsening of mobility, Infection at the stimulator site, Erroneous stimulator shut-off, Vertebral fracture from fall); Nonserious adverse event related to surgery(Subcutaneous seroma, Asymptomatic intracerebral hematoma, Postoperative confusion, Skin erosion, Extension cable discomfort, Pneumonia, Other adverse event); Nonserious adverse event related to stimulation or medication(Severe fluctuations in mobility, Dyskinesia, Dysarthria, Depression, Cognitive disturbances, Psychosis, Loss of affect, Other adverse events) |
|  |  | MT | Serious adverse event including Death(Car accident during psychotic episode) and Readmission to the hospital(Worsening of mobility, Hip fracture from fall); Nonserious adverse event related to stimulation or medication(Severe fluctuations in mobility, Dyskinesia, Psychosis, Other adverse events) |
| 36 | Anderson2005 | STN_DBS | NA |
|  |  | GPi_DBS | NA |
| 37 | Capecci2005 | STN_DBS | NA |
|  |  | MT | NA |
| 38 | Just2002 | STN_DBS | NA |
|  |  | MT | NA |
| 39 | Obeso2001 | STN_DBS | Related to procedure(Intracranial hemorrhage, Hemiparesis secondary to hemorrhage, Seizures, Infection, Improper lead placement, Brachial plexus injury, Confusion, Paralysis, Pulmonary embolus); Related to device(Migration, Infection, Lead break, Seroma, Erosion, Abnormal healing, Intermittent function); Related to stimulation(Dyskinesia, Diplopia, Accidental injury, Dysarthria, Headache, Paresthesia) |
|  |  | GPi_DBS | Related to procedure(Intracranial hemorrhage, Hemiparesis secondary to hemorrhage, Seizures, Dysarthria); Related to device(Migration, Infection, Lead break, Seroma);Related to stimulation(Dyskinesia, Dystonia, Abdominal pain) |
| *: medical treatment&sham procedure or off stimulation. NA: not available. | | | |
